# Immune cell topography of head and neck cancer

**DOI:** 10.1136/jitc-2024-009550

**Published:** 2024-07-24

**Authors:** Tara Muijlwijk, Dennis N L M Nijenhuis, Sonja H Ganzevles, Fatima Ekhlas, Carmen Ballesteros-Merino, Laura A N Peferoen, Elisabeth Bloemena, Bernard A Fox, Jos B Poell, C René Leemans, Ruud H Brakenhoff, Rieneke van de Ven

**Affiliations:** 1 Otolaryngology / Head and Neck Surgery, Amsterdam UMC Locatie VUmc, Amsterdam, The Netherlands; 2 Cancer Biology and Immunology, Cancer Centre Amsterdam, Amsterdam, The Netherlands; 3 Cancer Immunology, Amsterdam Institute for Immunology and Infectious Diseases, Amsterdam, The Netherlands; 4 Molecular and Tumor Immunology Laboratory, Providence Cancer Institute, Robert W. Franz Research Center at the Earle A. Chiles Research Institute, Portland, Oregon, USA; 5 Pathology, Amsterdam UMC - Locatie VUMC, Amsterdam, The Netherlands; 6 Maxillofacial Surgery/ Oral Pathology, Academic Center for Dentistry, Amsterdam, The Netherlands

**Keywords:** B cell, Head and Neck Cancer, Macrophage, T cell, Tumor microenvironment - TME

## Abstract

**Background:**

Approximately 50% of head and neck squamous cell carcinomas (HNSCC) recur after treatment with curative intent. Immune checkpoint inhibitors are treatment options for recurrent/metastatic HNSCC; however, less than 20% of patients respond. To increase this response rate, it is fundamental to increase our understanding of the spatial tumor immune microenvironment (TIME).

**Methods:**

In total, 53 HNSCC specimens were included. Using a seven-color multiplex immunohistochemistry panel we identified tumor cells, CD163+macrophages, B cells, CD8+T cells, CD4+T helper cells and regulatory T cells (Tregs) in treatment-naive surgical resection specimens (n=29) and biopsies (n=18). To further characterize tumor-infiltrating CD8+T cells, we stained surgical resection specimens (n=12) with a five-color tumor-resident panel including CD103, Ki67, CD8 and pan-cytokeratin. Secretome analysis was performed on matched tumor suspensions (n=11) to measure protein levels.

**Results:**

Based on CD8+T cell infiltrates, we identified four different immunotypes: fully infiltrated, stroma-restricted, immune-excluded, and immune-desert. We found higher cytokine levels in fully infiltrated tumors compared with other immunotypes. While the highest immune infiltrates were observed in the invasive margin for all immune cells, CD163+macrophages and Tregs had the highest tendency to infiltrate the tumor center. Within the tumor center, especially B cells stayed at the tumor stroma, whereas CD163+macrophages, followed by T cells, were more often localized within tumor fields. Also, B cells were found further away from other cells and often formed aggregates while T cells and CD163+macrophages tended to be more closely located to each other. Across resection specimens from various anatomical sites within the head and neck, oral cavity tumors exhibited the highest densities of Tregs. Moreover, the distance from B cells and T cells to tumor cells was shortest in oral cavity squamous cell carcinoma (OCSCC), suggesting more interaction between lymphocytes and tumor cells. Also, the fraction of T cells within 10 µm of CD163+macrophages was lowest in OCSCC, indicating fewer myeloid/T-cell suppressive interactions in OCSCC.

**Conclusions:**

We comprehensively described the TIME of HNSCC using a unique data set of resection specimens. We discovered that the composition, as well as the relative localization of immune cells in the TIME, differed in distinct anatomical sites of the head and neck.

**WHAT IS ALREADY KNOWN ON THIS TOPIC**

- We previously reported differences in the immune composition between anatomical sites of the head and neck using multiparametric flow cytometry. Until now, a comparison of the spatial tumor immune microenvironment in relation to the tumor site was lacking.

**WHAT THIS STUDY ADDS**

- This is the first study to describe the spatial cellular architecture of surgical resection specimens from various head and neck squamous cell carcinomas (HNSCC) anatomical sites, including the hypopharynx, reporting spatial differences alongside their deviating immune compositions.

**HOW THIS STUDY MIGHT AFFECT RESEARCH, PRACTICE OR POLICY**

- Our data encourages further research on cohorts treated with immune checkpoint inhibitors to link the spatial HNSCC tumor immune microenvironment to the response.

## Background

 Head and neck squamous cell carcinoma (HNSCC) has a poor prognosis with a recurrence rate of approximately 50% after treatment with curative intent, and an overall mortality rate of 51%.^1 2^ HNSCC originate in the mucosal lining along the upper aerodigestive tract with oral cavity SCC (OCSCC), larynx SCC (LSCC), oropharynx SCC (OPSCC) and hypopharynx SCC (HSCC) as most prevalent sites in Western countries. Risk factors for developing HNSCC are smoking, excessive alcohol consumption, genetic predisposition, and for tumors localized in the oropharynx, persistent and transforming infection with high-risk types of the human papillomavirus (HPV). HPV-positive and HPV-negative OPSCC are appreciated as distinct disease entities based on clinical and molecular characteristics, with an inferior prognosis for HPV-negative OPSCC: a 3-year overall survival of 57% compared with 82% for HPV-positive OPSCC.^3^

Depending on the tumor site and disease stage, HNSCCs are treated with surgery, (chemo)radiotherapy, or a combination of these modalities. Additionally, immune checkpoint inhibitors (ICIs) which target the interaction between programmed cell death 1 (PD-1) and its ligands, are treatment options for recurrent/metastatic HNSCC. Unfortunately, more than 80% of the patients do not respond to ICIs.^4 5^ The tumor immune microenvironment (TIME) plays an important role in prognosis and immunotherapy response, as it dictates the culmination of a suppressed or active antitumor immune response.^6^,^8^ It is therefore crucial to build a comprehensive understanding of the composition, as well as the topography of the TIME of HNSCC to ultimately improve response rates to ICIs.

The TIME consists of a variety of immune cells in and surrounding the tumor. Some of the key immune cells are tumor-infiltrating lymphocytes, with cytotoxic CD8+T cells as effector cells, responsible for the direct killing of tumor cells. Second, CD4+T helper cells are pivotal for activating CD8+T cells and B cells, but can by themselves also be cytotoxic. Regulatory T cells (Tregs) on the other hand, are known to suppress antitumor immune responses.^9 10^ The relation between Tregs and prognosis in HNSCC depends on their proximity to CD8+T cells. Specifically, a high number of Tregs within 30 µm of CD8+T cells is associated with a worse prognosis compared with a low number of Tregs around CD8+T cells.^8^ Over the past years, an increasing effort has been made to expand our understanding of the role of B cells in the TIME. B cells act as antigen-presenting cells, activate other immune cells in the TIME, such as T cells, produce tumor-specific antibodies, through which they can induce antibody-dependent cell-mediated cytotoxicity, and lastly, even directly induce the killing of tumor cells by producing granzyme B.^11^

Macrophages are abundantly present in the TIME and are known for their plasticity. Depending on environmental factors, they are polarized into a phenotype with antitumorigenic or protumorigenic features, often referred to as M1-like or M2-like macrophages, respectively. The latter can be recognized by markers such as CD163 or CD206. The presence of protumorigenic macrophages is described to be negatively correlated with prognosis in many cancers, including HNSCC.^12 13^ Chiu *et al* demonstrated that oral cancer cells can drive macrophages into a protumorigenic phenotype,^14^ and alternately, macrophages with M2-like features enhance tumor growth.^15^

Crosstalk between cells in the TIME is essential for a successful antitumor immune response. Cells within close proximity of each other, if not adjacent, are assumed to directly or indirectly interact. The exact location of cells within the TIME informs us of such interactions. In this study, we present wide-ranging spatial TIME analyses of treatment-naive HNSCC. We used a unique data set primarily comprised of surgical resection specimens, offering a comprehensive overview of the TIME topography in head and neck cancer across multiple anatomical sites.

## Methods

### Patients and specimens

Formalin-fixed paraffin-embedded (FFPE) tissue from primary tumors was obtained from a treatment-naive cohort of patients with HNSCC who underwent either a diagnostic biopsy or surgical excision of the tumor between 2019 and 2023 at Amsterdam UMC, location VUmc. HPV was routinely evaluated at the pathology department for diagnosis using p16 immunohistochemistry, and if p16 was positive, it was confirmed by HPV DNA testing.

### Multiplex immunohistochemistry opal staining

FFPE tissue sections of 3 µm were deparaffinized according to standard protocol followed by antigen retrieval (online supplemental methods). Next, they were manually immunostained with a seven-color panel including CD44v6, CD19, CD3, CD8, FoxP3, CD163 and DAPI. In addition, FFPE tissue sections were stained with a five-color panel including CD8, CD103, Ki67, pan-cytokeratin and DAPI using a Leica-Bond RX autostainer, as previously described.^16^ Antigen-antibody binding was visualized with tyramide signal amplification-Opal reagents (Akoya Biosciences). An overview of the staining methods, panel and antibodies can be found in online supplemental methods.

### Visualization and data analysis

Scanning was performed using the Vectra Polaris (PerkinElmer) or PhenoImager HT (Akoya Biosciences). Staining and scanning were executed in batches of approximately 10–20 slides. A scanning protocol was developed for each batch by calculating the median exposure time for all Opal markers, DAPI, and autofluorescence at multiple spots on one slide per batch. InForm V.2.6 was used for obtaining unmixed signals, and background staining was eliminated by using unstained negative controls.

QuPath V.0.4.3^17^ was used for image stitching, tissue annotation, cell detection, cell phenotyping, pixel classification, and distance measurements between individual cells. A trained head and neck pathologist reviewed the sections for tumor presence and assessment of histological parameters evaluated in the current study. Normal adjacent and dysplastic tissue was excluded from the analysis. Also, tissue with low-quality staining, such as the absence of DAPI or in case no separate cells were recognized, was excluded.

The spatial image analysis of tissue package V.1.4.1 was used to calculate average minimum distances (AMD) between cells.^18^ To identify immune cellular neighborhoods, imcRtools was used.^19^ Neighborhoods were defined by mapping all of the neighboring cells within 50 µm of each immune cell (CD163+macrophages, CD19+B cells, CD8+T cell, CD4+T helper and Tregs). Using k means unsupervised clustering, we distinguished four cellular neighborhoods per resection specimen. Cell densities were used as input for Pearson correlation analyses, executed using R V.4.2.3 and visualized using the package corrplot V.0.92. Samples with missing data were excluded from the analysis.

### Definitions

Diagnostic biopsies were obtained from the tumor center, whereas surgical resection specimens included the tumor center as well as the invasive margin. The tumor area was defined by all tumor tissue in one 3 µm resection slide, as scored by the pathologist, including stromal tissue in between tumor cells as well as surrounding stromal tissue till 250 µm from the outer tumor cells. Within the tumor area, tumor fields and tumor stroma were distinguished. This tissue segmentation was performed using a pixel classifier in QuPath, which was trained on CD44v6 staining as well as cell morphology. The tumor area was divided into the tumor center and invasive margin. The invasive margin was defined as the 500 µm outer layer of the tumor area, based on the definition by Pagès *et al*.^20^ In QuPath, the outer layer of tumor fields was manually outlined, followed by an automatically drawn radius of 250 µm. The tumor center was defined as the remaining tumor area.

### Secretome analysis

When fresh tumor material was available, matched treatment-naive tumor specimens were digested as previously described.^21^ In total 1×10^5^ single cells were cultured in 100 µL Roswell Park Memorial Institute 1640 medium (Lonza) supplemented with 10% fetal bovine serum (heat-inactivated FBS, Biological Industries), penicillin, streptomycin and L-glutamine (Lonza) at 37°C and 5% CO_2_. After 24 hours, 90 µL conditioned medium was collected, centrifuged for 5 min at 300 g and 80 µL was stored at −20°C until further use.

An Olink Target 96 proximity extension assay was performed on the overnight conditioned media to obtain normalized protein expression (NPX) levels of 92 proteins (online supplemental table 1). NPX is a unit on the log_2_-scale, used to compare the concentration of target proteins across samples.^22^ Three interplate controls were included per assay so that an interplate control normalization for each plate could be executed to reduce variation between plates. Moreover, an intensity normalization V.2 was performed in order to measure more plates at once. In brief, the intensity normalization V.2 adjusts the data so that the median NPX for a protein on each plate is equal to the overall median. Proteins were discarded from analysis when present in less than 85% of the samples. Clustering of the NPX values of the remaining 64 proteins was performed using R V.4.2.3.

### Statistical analysis

Statistical analyses were performed using GraphPad Prism V.9.5.1 and R V.4.2.3. Paired data were tested by a two-sided signed-rank test and unpaired data were tested by a two-sided rank-sum test. Paired data in multiple groups were tested by the Friedman test. Unpaired data with multiple groups were tested by the Kruskal-Wallis test. The significance of groups was assessed by uncorrected Dunn’s tests. Χ^2^ test was used for the analysis of contingency tables with multiple categories. Correlations were assessed using Spearman’s rank correlation coefficient.

## Results

### Immunotypes of HPV-negative HNSCC defined by CD8+ T-cell densities

**Figure 1:**
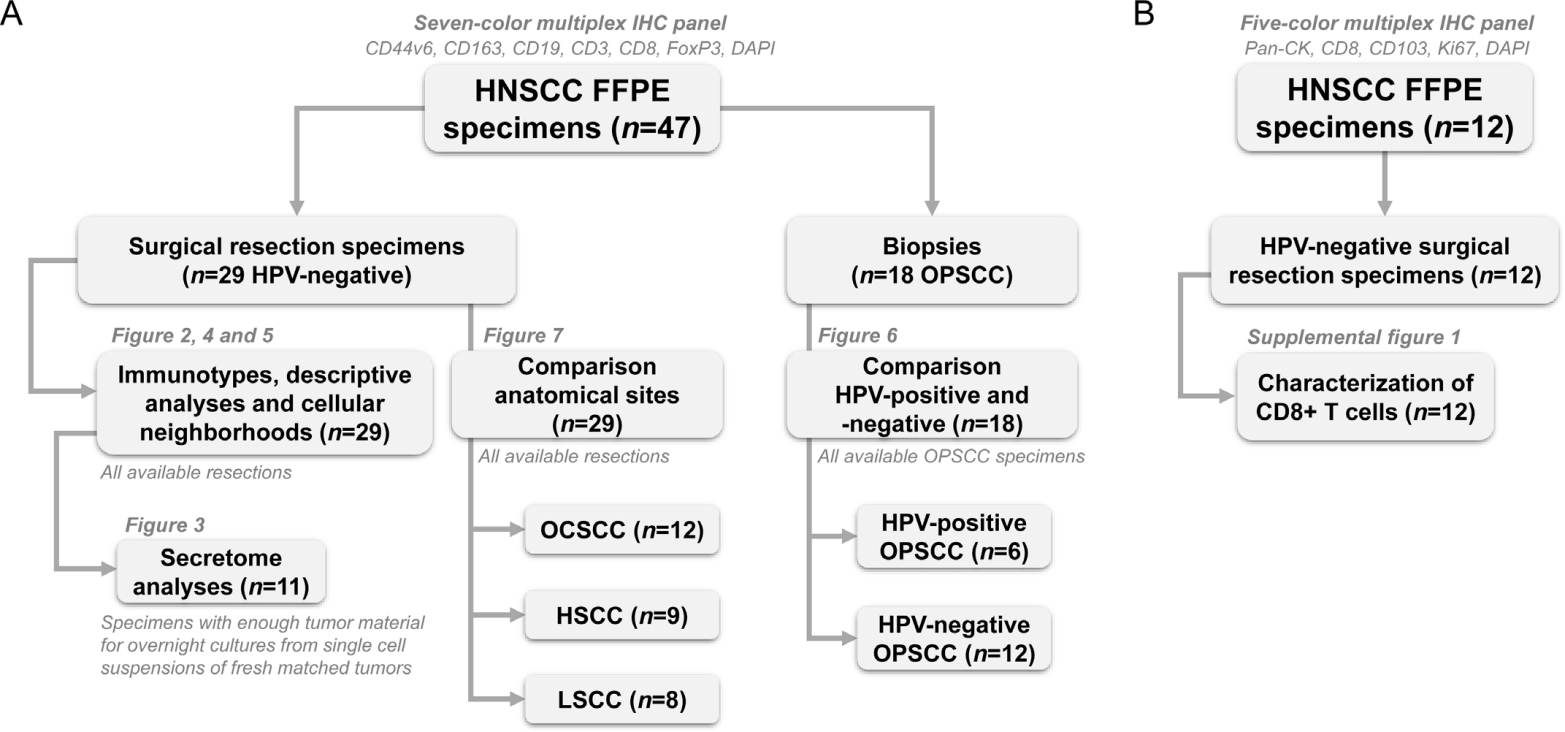
Flowchart of head and neck squamous cell carcinoma (HNSCC) cohort used in this study. (**A**) 47 HNSCC formalin-fixed paraffin-embedded (FFPE) specimens were stained using a seven-color multiplex immunohistochemistry (IHC) panel to distinguish CD44v6+tumor cells, CD163+macrophages, CD19+B cells, CD8+T cells, CD3+CD8− FoxP3− T cells (CD4+T helper cells) and FoxP3+regulatory T cells. 29 out of those 47 were HPV-negative surgical resection specimens. These resection specimens were used for assigning immunotypes (figure 2), descriptive analyses (figure 4), and immune cellular neighborhoods (figure 5). For 11 out of the 29 tumors, secretome data was available of overnight cultures from matched fresh single-cell suspensions (figure 3). All 29 resection specimens were used to compare the TIME from different anatomical sites: OCSCC (n=12), hypopharynx SCC (HSCC, n=9) and larynx SCC (LSCC, n=8, figure 7). Lastly, 18 out of 47 HNSCC FFPE specimens were oropharynx SCC (OPSCC) biopsies for the comparison between the TIME of HPV-positive (n=6) and HPV-negative (n=12) OPSCC (figure 6). (**B**) 12 HNSCC FFPE specimens were stained using a five-color multiplex IHC panel to distinguish pan-cytokeratin (pan-CK)+tumor cells, CD103+tumor-resident, CD103− recruited, Ki67+proliferating and Ki67− non-proliferating CD8+T cells. All 12 resections were used for the characterization of infiltrating CD8+T cells (online supplemental figure 2). 6 out of those 12 specimens overlapped between the seven-color and five-color multiplex IHC panels, explaining the total number of 53 unique specimens. HHPV, human papillomavirus; OCSCC, oral cavity SCC; TIME, tumor immune microenvironment.

**Figure 2:**
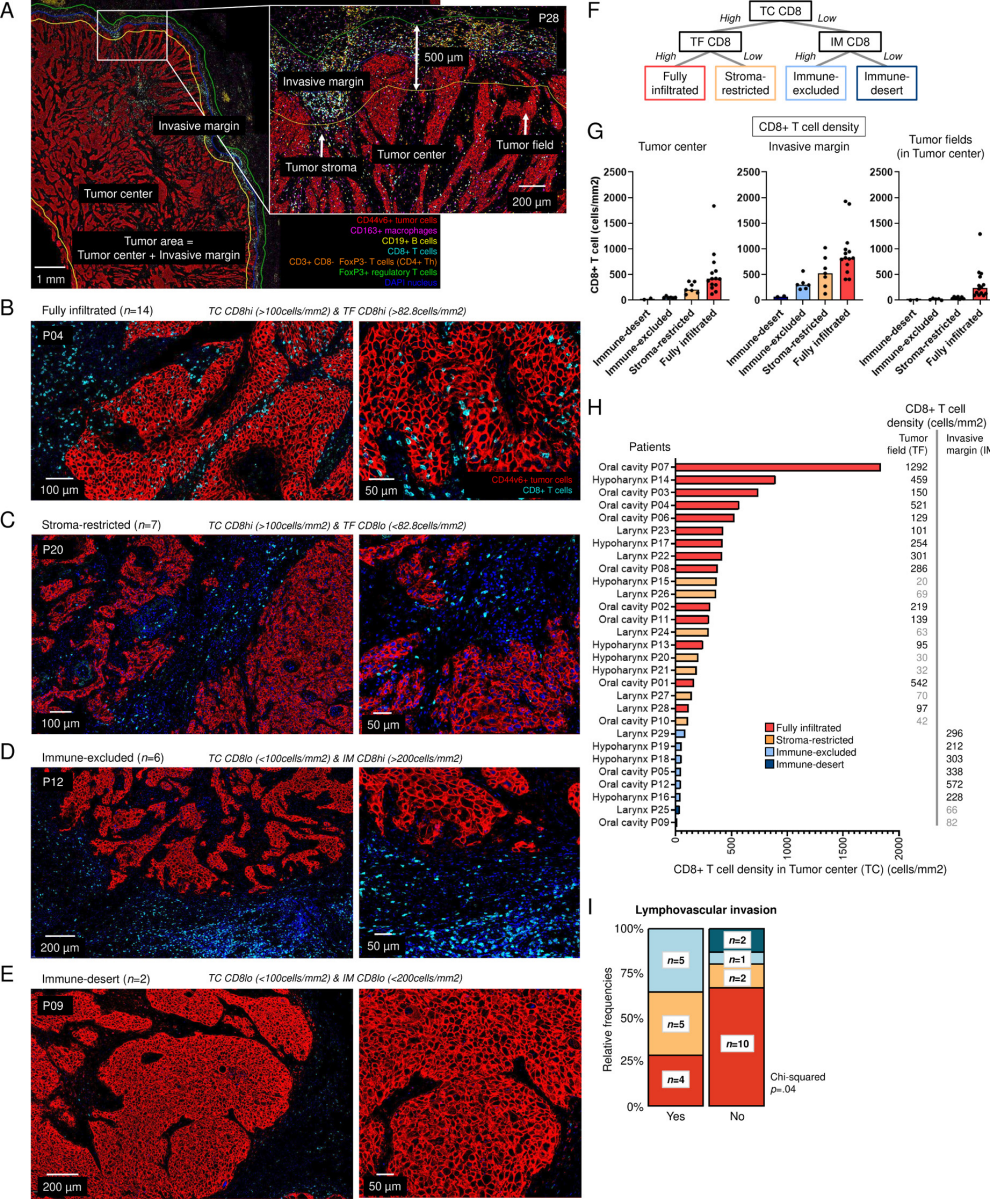
Immunotypes across 29 human papillomavirus-negative head and neck squamous cell carcinoma (HNSCC) resection specimens. (**A**) Representative image of tumor P28 with tumor center, invasive margin, tumor fields and tumor stroma. The following cells could be distinguished: CD44v6+tumor cells, CD163+macrophages, CD19+B cells, CD8+T cells, CD3+CD8 T cells (CD4+T helper cells) and FoxP3+regulatory T cells. (**B–E**) Representative images of tumors with (**B**) fully infiltrated (P04), (**C**) stroma-restricted (P20), (**D**) immune-excluded (P12), and (**E**) immune-desert (P09) immunotype. (**F**) Decision tree for assigning immunotypes and immunotypes based on CD8+T cell density in tumor center, invasive margin and tumor fields. (**G**) CD8+T cell densities (cells/mm^2^, y-axis) across immune-desert, immune-excluded, stroma-restricted and fully infiltrated HNSCC (x-axis). (**H**) 29 HNSCC resection specimens were assigned as infiltrated when the density of CD8+T cells in the tumor center (TC, x-axis) was higher than 100 cells/mm^2^. If the density of CD8+T cells in the tumor field (TF) was higher than the median density of 82.8 cells/mm^2^, tumor were assigned as fully infiltrated (red) and when lower than 82.8 cells/mm^2^, as stroma-restricted (orange). If the density was lower than 100 cells/mm^2^ in the tumor center but higher than 200 cells/mm^2^ in the invasive margin (IM), tumors were defined as immune-excluded (light blue) and when lower than 200 cells/mm^2^, as immune-desert (dark blue). (**I**) Relative frequencies and number of specimens per immunotype with the presence of lymphovascular invasion. Χ^2^ test was performed to obtain p value. Fully infiltrated immunotype compared with other immunotypes since groups were too small.

The HNSCC cohort used in this study is summarized as a flowchart in figure 1. Detailed patient and tumor characteristics can be found in onlinesupplemental tables 2  3. Using CD8+T cell densities, we assigned immunotypes to 29 HPV-negative HNSCC resection specimens (figure 2). Definitions were based on CD8+T cell densities in the tumor center, tumor fields, and invasive margin, as reported by Gruosso *et al* in breast cancer^23^ and reviewed by Tiwari *et al*.^24^ 14 tumors (48%) were assigned as inflamed, fully infiltrated tumors, defined by tumors with a CD8+T cell density in the tumor center of more than 100 cells/mm^2^ as well as a density in tumor fields of more than the median of 82.8 cells/mm^2^ (figure 2B). Seven tumors (24%) were specified as inflamed stroma-restricted: while those had a CD8+T cell density in the tumor center higher than 100 cells/mm^2^, CD8+T cells tended to reside in tumor stroma with a density in tumor fields of less than the median of 82.8 cells/mm^2^ (figure 2C). Six tumors (21%) were assigned as immune-excluded with less than 100 CD8+T cells/mm^2^ in the tumor center but a density of higher than 200 CD8+T cells/mm^2^ in the invasive margin (figure 2D). Lastly, two tumors (7%) were assigned as immune-desert, with barely any CD8+T cells in both the tumor center and the invasive margin (figure 2E). Immunotypes were independent of tumor characteristics such as the presence of desmoplastic stroma reaction, invasion pattern, differentiation grade, T-stage, disease-stage, recurrences or presence of extranodal extension (online supplemental figure 1). However, interestingly, fully infiltrated HNSCCs less often (4/14 29%) showed lymphovascular invasion compared with other immunotypes (10/15 67%, p=0.04, figure 2I).

### Higher proportion proliferating CD8+ T cells in tumor fields versus tumor stroma in HPV-negative HNSCC

We used a five-color multiplex immunohistochemistry (IHC) Opal panel to distinguish tumor-resident (CD103+), recruited (CD103–), proliferating (Ki67+) and non-proliferating (Ki67−) CD8+T cells within tumor stroma and tumor fields of 12 HPV-negative HNSCC resection specimens (figure 1B, online supplemental figure 2). While the majority of CD8+T cells in the tumor stroma were recruited, they were predominantly tumor-resident in tumor fields (60% and 58%, respectively, online supplemental figure 2B). Furthermore, the fraction of recruited as well as tumor-resident proliferating (Ki67+) CD8+T cells was higher in tumor fields compared with tumor stroma (7% vs 3%, p=0.015 and 14% vs 7%, p=0.002, respectively, online supplemental figure 2B, C).

### Higher cytokine levels in fully infiltrated tumors compared with tumors with other immunotypes

**Figure 3:**
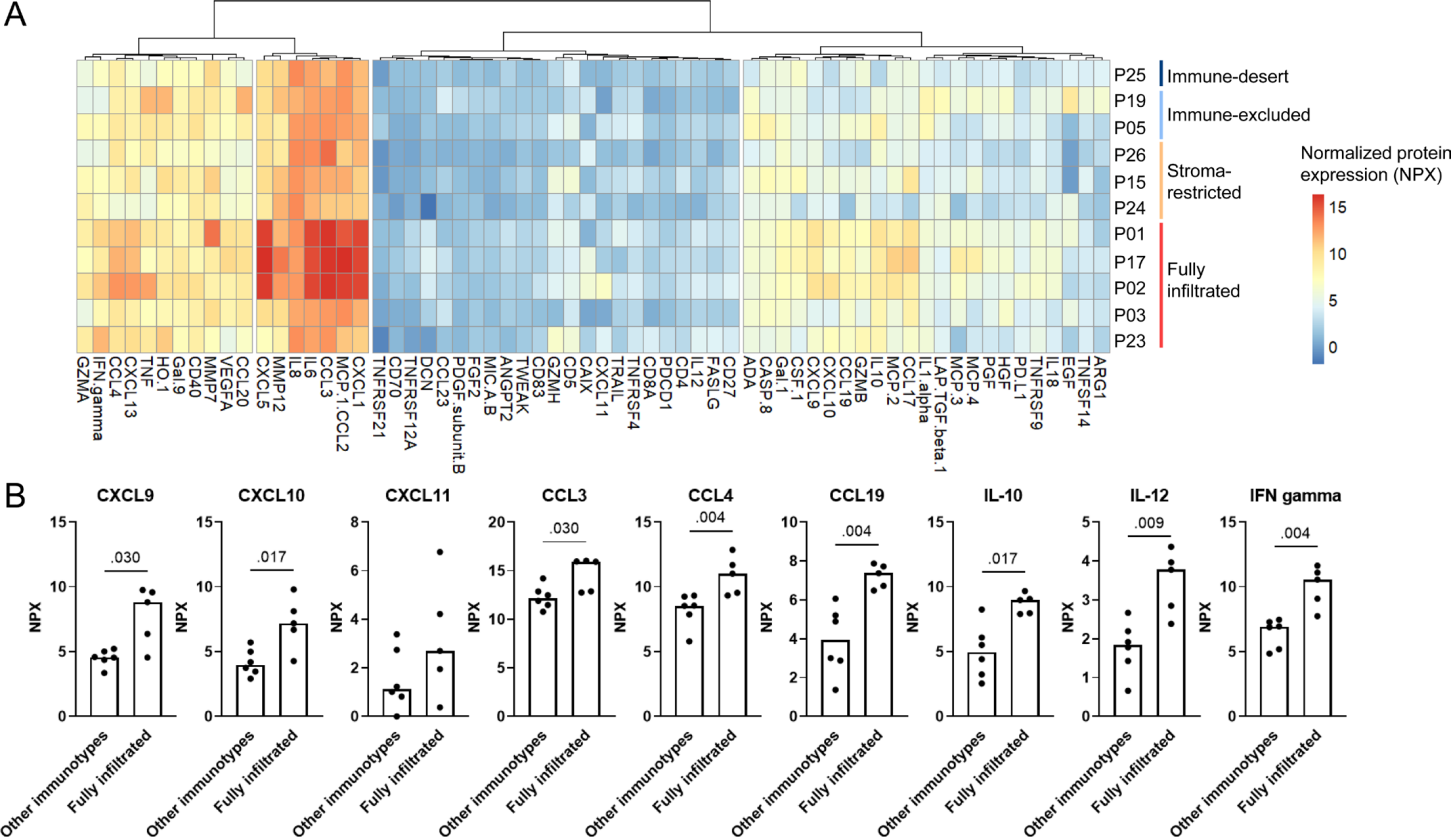
Immunotypes and secretomes across 11 HPV-negative head and neck squamous cell carcinoma resection specimens. (**A**) Hierarchical clustering of normalized protein expression (NPX) levels of 64 proteins (x-axis) measured in overnight supernatants of matched fresh single-cell suspensions from 11 tumors (y-axis). (**B**) Secretome comparison between fully infiltrated tumors versus other immunotypes. Protein levels (in normalized protein expression (NPX) values) measured in overnight cultures of 100,000 single cells from matched tumors on y-axis, in fully infiltrated tumors (n=5) and tumors with other immunotypes (immune-desert n=1, immune-excluded n=2 and stroma-restricted n=3) on x-axis, p values obtained by unpaired non-parametric Mann-Whitney tests, bars represent median. CCL, CC motif chemokine ligand; CXCL, C-X-C motif chemokine ligand; IFN, interferon; IL, interleukin.

Next, we examined whether we could link the secretome of overnight single-cell suspensions from 11 matched tumors to their immunotype (figure 3). Interestingly, fully infiltrated tumors showed higher cytokine levels compared with tumors with stroma-restricted, immune-excluded or immune-desert immunotypes (figure 3). As C-X-C motif chemokine ligand (CXCL)-9, CXCL-10, CXCL1-11, CC motif chemokine ligand (CCL)-3, CCL-4, CCL19, interleukin (IL)-10, IL-12 and interferon-γ are described to either directly or indirectly attract or provide a positive survival signal for T cells,^25 26^ we examined their NPX levels separately (figure 3B).

We investigated whether immune cell densities correlated with protein levels (online supplemental figure 3). Interestingly, the density of Tregs in the tumor area positively correlated with levels of CCL17 in the secretome of matched tumors (Spearman’s ρ=0.70, p=0.02). Of note, no reverse correlations were found between suppressive cytokines (such as IL-10 or vascular endothelial growth factor) and immune cell densities.

### Immune cells predominantly reside in the invasive margin of HPV-negative HNSCC

**Figure 4:**
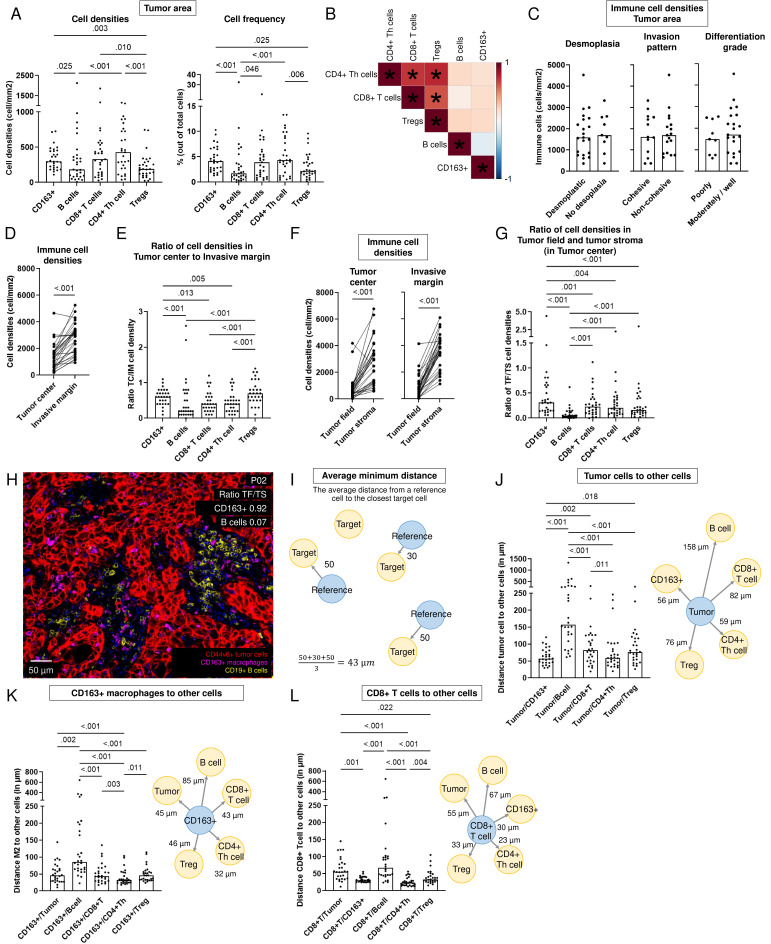
Spatial tumor immune microenvironment of 29 HPV-negative head and neck squamous cell carcinoma (HNSCC) resection specimens. (**A**) Densities (in cells/mm^2^) and frequency of total cells in tumor area of CD163+macrophages, B cells, CD8+T cells, CD4+T helper cells and Tregs. A paired non-parametric Friedman test was performed with uncorrected Dunn’s test to obtain p values. Bars represent the median. (**B**) Pearson correlation matrix with correlation coefficient from −1 (blue) to 1 (red) of immune cell densities in tumor area of 29 HPV-negative HNSCC resection specimens. P values<0.05 indicated with *. (**C–D**) Immune cell density (in cells/m^2^, y-axis) (**C**) across histological parameters (x-axis), p values obtained by an unpaired non-parametric Mann-Whitney test and (**D**) in the tumor center versus the invasive margin (x-axis), p value obtained by a paired non-parametric Wilcoxon test. (**E**) Ratio of immune cell density between tumor center (TC) and invasive margin (IM) for CD163+macrophages, B cells, CD8+T cells, CD4+T helper cells and Tregs. A paired non-parametric Friedman test was performed with uncorrected Dunn’s test to obtain p values. (**F**) Immune cell density (in cells/m^2^, y-axis) in tumor fields and tumor stroma (x-axis) of the tumor center (left) and in the invasive margin (right), p value obtained by a paired non-parametric Wilcoxon test. (**G**) Ratio of immune cell density between tumor field (TF) and tumor stroma (TS) in the tumor center for CD163+macrophages, B cells, CD8+T cells, CD4+T helper cells and Tregs. A paired non-parametric Friedman test was performed with uncorrected Dunn’s test to obtain p values. Bars represent the median. (**H**) Representative image with CD163+macrophages infiltrating into tumor fields and B cells predominantly in the tumor stroma example of tumor with a ratio CD163+macrophages in tumor field to tumor stroma of 0.92 and a ratio B cells in tumor field to tumor stroma of 0.07 (P02). (**I**) Schematic overview of calculation of the average minimum distance from a reference cell (in blue) to the nearest target cells (in yellow). (**J–L**) Distance (in µm, y-axis) of (**J**) Tumor cells, (**K**) CD163+macrophages and (**L**) CD8+T cells to other cells in the tumor area (x-axis) in left panels. A paired non-parametric Friedman test was performed with uncorrected Dunn’s test to obtain p values. Bars represent the median. A schematic overview in the right panel with distance from reference to the target cell, median over 29 resection specimens shown. HPV, human papillomavirus; Treg, regulatory T cells.

We examined the composition and distribution of immune cells within 29 HPV-negative HNSCC resection specimens (figure 4). Major differences were observed in immune cell densities and frequencies across tumors (figure 4A). Overall, CD4+T helper cells and CD8+T cells were most abundant among the immune cells investigated (figure 4A, online supplemental figure 4A). Moreover, CD4+T helper and CD8+T cell densities correlated significantly with Treg densities. Interestingly, no negative correlations were found between CD163+macrophages and T-lymphocyte or B-lymphocyte densities (figure 4B).

**Figure 5:**
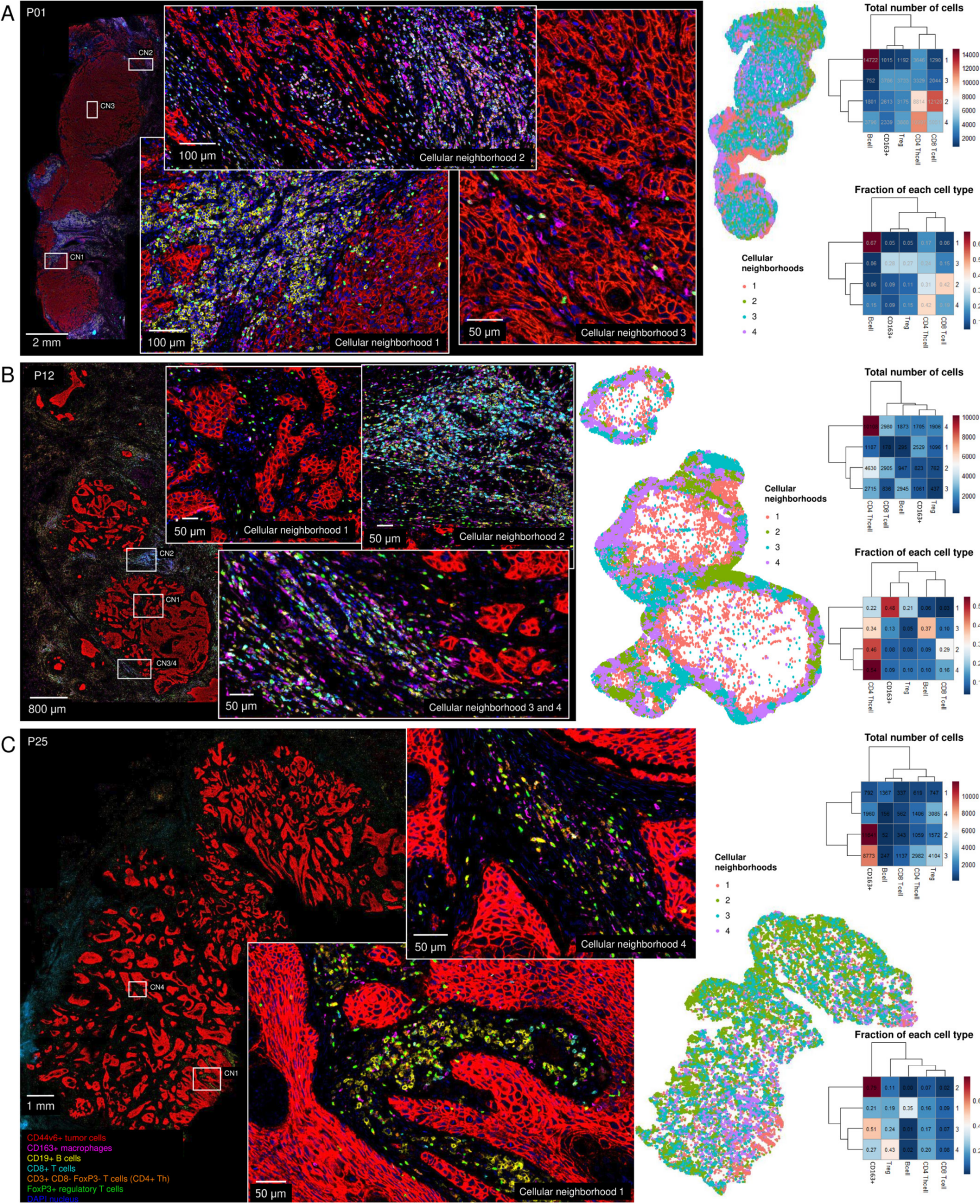
Immune cellular neighborhood analysis for 29 human papillomavirus-negative head and neck squamous cell carcinoma resection specimens. Neighboring cells in a radius of 50 µm were calculated using imcRtools.^19^ For each tumor, four neighborhoods were calculated. (**A–C**) Representative images of tumors from (**A**) P01, (**B**) P12, and (**C**) P25 with an animated map of immune cellular neighborhoods, a heatmap with total number of cells per neighborhood and a heatmap with the fraction of each cell type per neighborhood (blue to red, y-axis) per cluster (x-axis). Neighborhood analysis of remaining tumors can be found in online supplemental figures 8 and 9. Treg, regulatory T cells.

Immune cell density was independent of histological characteristics of the tumor, such as the presence of desmoplastic stroma reaction, invasion pattern or differentiation grade (figure 4C). In addition, we examined whether the TIME differed in regard to T-stage, N-stage, sex, age and smoking status. While no differences in immune cell densities were found in terms of tumor size (T2 and T3 vs T4), nodal involvement (N0 and N1 vs N2 and N3), sex, or smoking status, we found more immune infiltration for patients younger than the median age of HPV-negative tumors in this cohort, 68 years, compared with those who were older (online supplemental figure 5). Specifically, younger patients exhibited higher CD8+T cell densities (median of 474 vs 128 cells/mm^2^, p=0.002), CD4+T helper cell densities (median of 725 vs 285 cells/mm^2^, p=0.004) and Treg densities (median of 254 vs 126 cells/mm^2^, p<0.001, online supplemental figure 5A). Consistently, T-cell densities negatively correlated with age at diagnosis, while this correlation was evidently not observed for CD163+macrophages or B cells (online supplemental figure 5B).

For the majority of tumors (83%), immune cells predominantly resided in the invasive margin (p<0.001, figure 4D, online supplemental figure 4C). Only for one tumor (3%), the density of total immune cells was higher in the tumor center compared with the invasive margin (online supplemental figure 4E), whereas densities were comparable for four tumors (14%, online supplemental figure 4D). Tregs and CD163+macrophages had a higher tendency to infiltrate into the tumor center compared with B cells, CD8+T cells and CD4+T helper cells (figure 4E).

For the whole tumor area, both in the tumor center as well as in the invasive margin, immune cells predominantly resided in tumor stroma and were found in lower densities within tumor fields (figure 4F, online supplemental figure 6A, B). For only one tumor (P01, 3%), a higher density of immune cells was found in tumor fields compared with tumor stroma (online supplemental figure 6C). While the tendency to reside in tumor stroma was observed for all immune cell types studied, the contrast was particularly noticeable for B cells (figure 4G,H).

### CD163+ macrophages and T cells localize near tumor cells and each other while B cells are more isolated in HPV-negative HNSCC

We analyzed the location of the tumor and immune cells relative to each other using the AMD (figure 4I–L, online supplemental figure 7). B cells appeared to be located the furthest away from tumor cells as well as from other immune cells, explained by a more clustered localization of B cells in the tumor (online supplemental figure 7E). CD163+macrophages were the immune cells located closest to tumor cells. Noteworthy, CD8+T cells were located further away from tumor cells compared with CD163+macrophages (82 µm vs 56 µm, respectively, p=0.002, figure 4J). CD8+T cells, CD4+T helper cells, and Tregs were closest located to each other (CD8+T cells to CD4+T helper cells 23 µm and to Tregs 33 µm) and to macrophages (30 µm), while tumor cells were situated further away (55 µm), and B cells even more so (67 µm, figure 4L).

### Immune cellular neighborhoods in HPV-negative HNSCC

To examine which immune cell types co-localize, we performed immune cellular neighborhood analysis for 29 HPV-negative resection specimens (figure 5, online supplemental figures 8 and 9). Neighborhoods were defined by mapping all of the neighboring cells within 50 µm of each immune cell using imcRtools.^19^ Using k means unsupervised clustering, we distinguished four cellular neighborhoods per tumor.

T cells often co-localize with each other. Most of the time, CD4+T helper cells and CD8+T cells did not dominate a single neighborhood, and comprised less than 40% of a cellular neighborhood (in 20 and 16 out of 29 tumors, respectively). However, in some tumors, neighborhoods were found dominated (present in ≥40% of a cluster) by CD4+T helper cells (P08, P10-P12), CD8+T cells (P03, P07, P17, P20, P22, P24, P26, P29), or by both (P01, P04, P14, P16, P23).

Importantly, some tumors assigned as immune-excluded immunotypes, based on CD8+T cell densities (figure 2), displayed CD4+T helper cell infiltration into the tumor center (P12, P16). In addition, immune-desert immunotypes appeared not to be entirely deserts since they displayed immune cellular neighborhoods containing other immune cells. By way of illustration, P09 showed B cell and CD163+macrophages dominated neighborhoods, not restricted to the invasive margin but also located in the tumor center. Second, immune-desert tumor P25 exhibited CD4+T helper cell and B-cell neighborhoods (figure 5C). Note that while CD8+T cell densities were logically highest in fully infiltrated tumors, this was not the case for CD163+macrophages and B cells (online supplemental figure 10). CD163+macrophages were evenly high in all immunotypes.

Tregs spread out very evenly through the tumor and infiltrated the tumor center. In the same line, neighborhoods with CD163+macrophages were located across the entire tumor area, in the invasive margin as well as in the tumor center. Only for one tumor, CD163+macrophages seemed limited to the invasive margin (P05). Neighborhoods located in the tumor center were quite heterogeneous with various cell types present, however, often Tregs (P05, P16), CD163+macrophages (P09, P12, P13, P16) alone or together (P18-P20, P25, P29) were mostly present in neighborhoods localized in the tumor center.

B cells were typically localized as aggregates (seen in 19 out of 29 tumors, 66%), limited to the invasive margin (8 out of 19 tumors, 42%), or also in the tumor center (11 out of 19 tumors, 58%). Most commonly, B-cell aggregates were identified (online supplemental figure 11); however, for two tumors (P13, P24) they seemed more like an organized tertiary lymphoid structure (TLS, online supplemental figures 12 and 13). Nonetheless, we cannot ascertain whether they were TLS as we did not include the necessary markers for identification in our panel.^27^

Generally, the immune cellular neighborhoods were spread out through the tumor area. However, sometimes a clear separation was observed between neighborhoods localized in the invasive margin versus the tumor center (P01, P05, P08, P09, P12, P16-P18, P23), or between two parts of the tumor, independently of the invasive margin and tumor center (P15, P20, P22), indicating tumor heterogeneity in terms of immune infiltration. Note that in some cases annotating the tumor area was difficult since tumor field islands far from the tumor core were present (P12, P20). Overall, each HNSCC resection specimen had its own immune composition with immune cellular neighborhoods appearing.

### Higher B-lymphocyte and T-lymphocyte densities in HPV-positive oropharyngeal tumors

**Figure 6:**
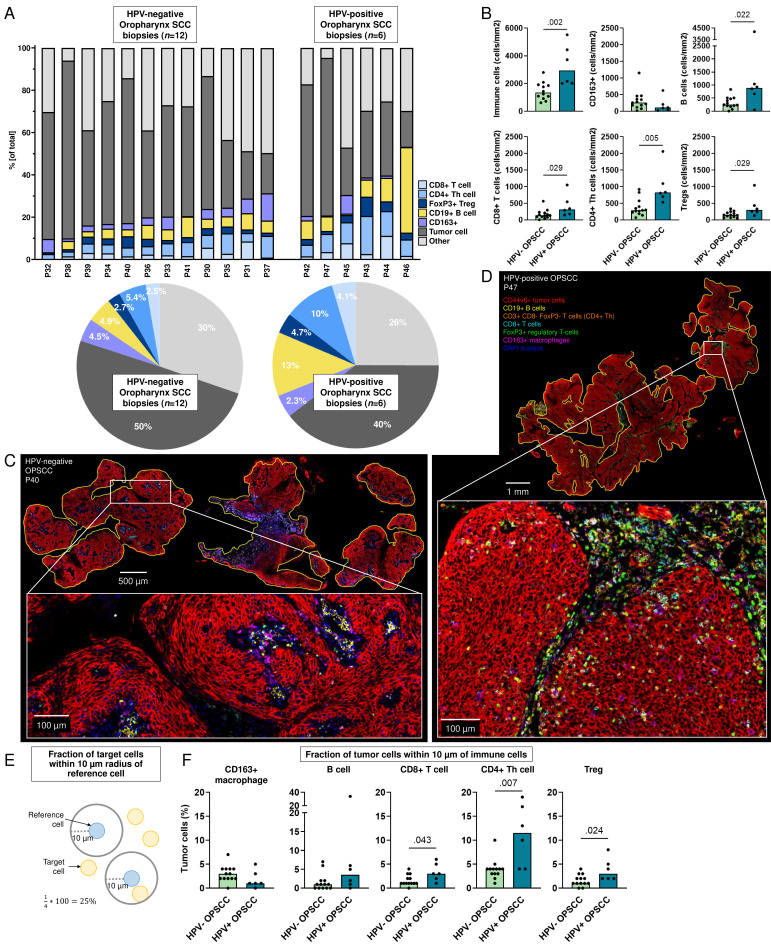
Spatial tumor immune microenvironment of HPV-negative and HPV-positive oropharynx SCC (OPSCC). (**A**) Fraction of tumor and immune cells out of total cells (y-axis) in biopsies from patients with HPV-negative and HPV-positive OPSCC (x-axis). Pie charts of the average percentage of cells in 12 HPV-negative (left panel) and 6 HPV-positive OPSCC biopsies. (**B**) Densities of immune cells (cells/mm^2^, y-axis) in HPV-negative and HPV-positive OPSCC (x-axis), p values obtained by unpaired non-parametric Mann-Whitney tests, bars represent median values. (**C–D**) Representative image of (**C**) HPV-negative (P40) and (**D**) HPV-positive (P47) OPSCC stained with seven-color multiplex immunohistochemistry Opal panel to identify CD44v6+tumor cells, CD163+macrophages, CD19+B cells, CD8+T cells, CD3+CD8− FoxP3− (CD4+T helper) cells and FoxP3+regulatory T cells (Tregs). (**E**) Schematic representation of radius analysis where the fraction of target cells (in yellow) located within 10 µm of a reference cell (in blue) was calculated. (**F**) Percentage of tumor cells (y-axis) within 10 µm of CD163+macrophage, B cell, CD8+T cell, CD4+T helper (Th) cell and Treg for 12 HPV-negative (HPV−) and 6 HPV-positive (HPV+) biopsies, p values obtained by unpaired non-parametric Mann-Whitney tests, bars represent median values. HPV, human papillomavirus; SCC, squamous cell carcinoma.

Since oropharyngeal tumors are primarily treated by definitive radiotherapy or chemoradiotherapy and not surgery,^28^ it is impossible to obtain resection specimens. Consequently, we only had access to biopsies for the comparison of the TIME of HPV-positive and HPV-negative OPSCC (figure 6). The TIME of HPV-positive and negative OPSCCs evidently differed with higher immune cell densities in HPV-positive biopsies, explained by greater tumor-infiltrating B and T cells (figure 6A–D). Conversely, HPV-negative OPSCCs showed higher densities of CD163+macrophages (mean 316 cells/mm^2^ vs 183 cells/mm^2^, respectively), although this was not significant (p=0.152, figure 6B). Lastly, the percentage of tumor cells located within 10 µm of a T cell was higher in HPV-positive compared with HPV-negative OPSCCs (figure 6E,F).

### Highest T-cell densities in tumors originating from the oral cavity compared with the larynx and hypopharynx

**Figure 7:**
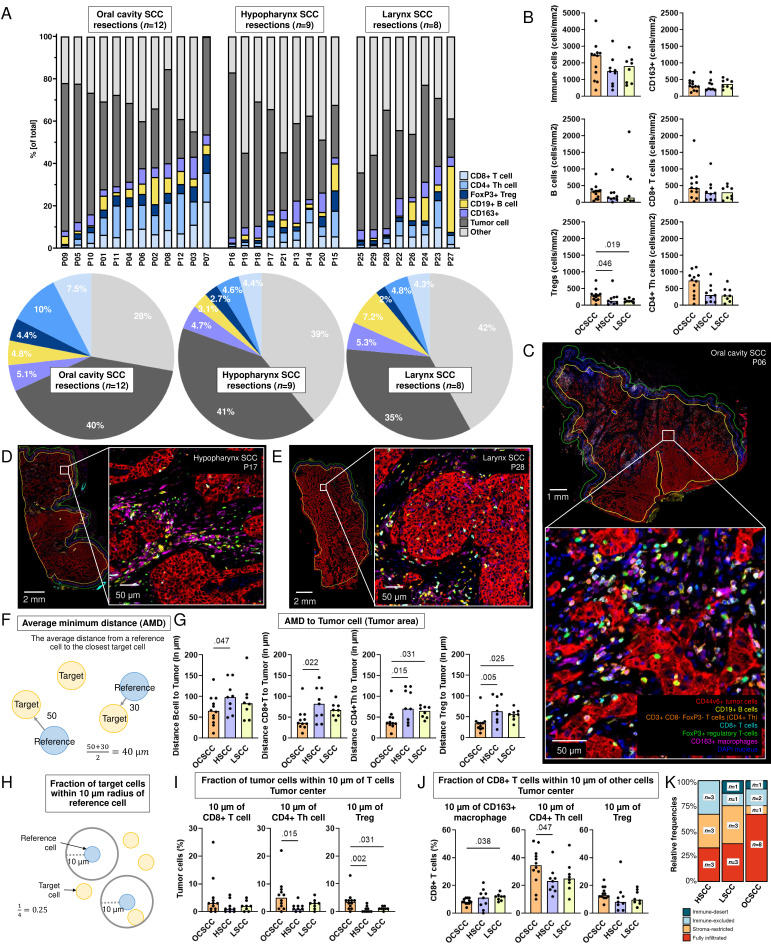
Comparison of 29 human papillomavirus-negative resection specimens from distinct anatomical sites along the head and neck region. (**A**) Fraction of tumor and immune cells out of total cells (y-axis) in tumor area of surgical resection specimens from patients with oral cavity squamous cell carcinoma (OCSCC, n=12), hypopharynx SCC (HSCC, n=9), and larynx SCC (LSCC, n=8, x-axis). Pie charts of the average percentage of cells in the tumor area of 12 OCSCC (left panel), 9 HSCC (middle panel), and 8 LSCC (right panel) surgical resection specimens. (**B**) Densities of immune cells in tumor area (cells/mm^2^, y-axis) of OCSCC, HSCC and LSCC (x-axis). (**C–E**) Representative images of (**C**) OCSCC (P06), (**D**) HSCC (P17), and (**E**) LSCC (L28) stained with seven-color multiplex immunohistochemistry Opal panel to identify CD44v6+tumor cells, CD163+macrophages, CD19+B cells, CD8+T cells, CD3+CD8− FoxP3− (CD4+T helper (Th)) cells and FoxP3+regulatory T cells (Tregs). (**F**) Schematic overview of calculation of average minimum distance (AMD) from a reference cell (in blue) to the nearest target cells (in yellow). (**G**) Distance (in µm, y-axis) of B cells, CD8+T cells, CD4+Th cells, and Tregs to tumor cells in the tumor area from OCSCC, HSCC, and LSCC resection specimens (x-axis). (**H**) Schematic representation of radius analysis where the fraction of target cells (in yellow) located within 10 µm of a reference cell (in blue) was calculated. (**I**) Fraction of tumor cells (y-axis) within 10 µm of CD8+T cell, CD4+Th cell, and Treg in tumor center of OCSCC, HSCC, and LSCC resections. (**J**) Fraction of CD8+T cells (y-axis) within 10 µm of CD163+macrophage, CD4+Th cell, and Treg in tumor center of OCSCC, HSCC, and LSCC resections. Unpaired non-parametric Kruskal-Wallis tests with uncorrected Dunn’s tests were performed to obtain p values, bars represent median values. (**K**) Relative frequencies and number of specimens (y-axis) classified as fully infiltrated (red), stroma-restricted (orange), immune-excluded (light blue), or immune-desert (dark blue) per anatomical site (x-axis).

We previously reported differences in the immune composition between anatomical sites using multiparametric flow cytometry.^21^ Therefore we here compared the spatial TIME of 29 surgical resection specimens originating from the oral cavity (n=12), hypopharynx (n=9), and larynx (n=8, figure 7). While interpatient heterogeneity was observed in the immune composition of tumors, OCSCCs demonstrated higher levels of T-cell infiltrates, which could be attributed to higher densities of Tregs, in comparison to HSCC and LSCC (p=0.046, p=0.019, figure 7A–D). No differences were found in the presence of desmoplastic tissue, invasion pattern or differentiation grade across anatomical sites (online supplemental figure 14), but noteworthy, in OCSCCs, 8 out of 12 tumors (67%) were assigned as fully infiltrated immunotypes based on their CD8+T cell infiltrates, while this was 3 out of 9 (33%) and 3 out of 8 (38%) for HSCC and LSCC, respectively (figure 7K).

Interestingly, the average minimum distance from B cells, CD8+T cells, CD4+T helper cells, and Tregs to tumor cells was shortest in tumors originating from the oral cavity (figure 7G, online supplemental figure 15A, B). In accordance with this, more tumor cells were found within a 10 µm radius of T cells (figure 7I, online supplemental figure 15C, D). The relatively closer proximity of lymphocytes to tumor cells, as well as the proximity between B and T cells, suggests more cellular interaction in OCSCC among B and T cells, as well as with tumor cells. When comparing the percentages CD8+T cells and of CD4+T helper cells within a 10 µm radius of CD163+macrophages, this was the lowest for OCSCCs (figure 7J, online supplemental figure 16), indicating less interaction of CD163+macrophages with T cells in tumors originating from the oral cavity and possibly less immune suppression by CD163+macrophages. Lastly, the higher fraction of CD8+T cells within 10 µm of CD4+T helper cells (figure 7J) indicates more interaction between T cells in OCSCC.

## Discussion

In this study, we investigated the spatial TIME of head and neck cancers using a unique data set of resection specimens from various anatomical sites. Current multiparametric IHC studies in the context of HNSCC are either focused on differences between HPV-positive and HPV-negative OPSCCs,^29^,^31^ or comprehensively describe the TIME, but in small cohorts,^32^ or describe a large cohort of (primarily) OCSCCs.^8 33 34^ Except for studies involving OCSCCs,^8 33 34^ often regions of interest or biopsies are used for TIME analysis instead of surgical resection specimens.^29^,^32^

Since HSCCs are the least common among the HNSCC,^28^ data describing the TIME of HSCC are scarce. To our knowledge, we are the first to present multiparametric spatial data on this anatomical site in comparison to OCSCC and LSCC. We demonstrated that the TIME of HSCC clearly differed from that of tumors originating from the oral cavity. It seemed that the immune composition of HSCC was more comparable with LSCC, which might be explained by the anatomically closer proximity. Interestingly, in concordance with our work using multiparameteric flow cytometry on fresh HNSCC single-cell suspensions,^21^ we found higher CD4+T cell densities in tumors originating from the oral cavity compared with HSCC and LSCC. Of note, patients from that study^21^ and the current (partly) overlap (online supplemental table 2), but a different tumor sample was examined. Additionally, a different technique was employed to investigate the TIME, each with its own advantages and limitations. At present, we have no explanations for these higher CD4+T cell levels in OCSCCs. It remains speculation whether the oral microbiome plays a role.

We identified the majority of HNSCC as either being fully infiltrated (48%) or stroma-restricted (24%). In other words, in 72% of the surgical resections investigated, CD8+T cells infiltrated into the tumor center. As HNSCCs are known for their low response rate to ICIs, this implies that even though CD8+T cells might be present in the tumor, immunosuppressive factors hamper an effective antitumor immune response, such as protumoral macrophages. For some tumors, hardly any CD8+T cell infiltration was observed (7%), or CD8+T cells were located solely in the invasive margin and did not enter the tumor center (21%). Those immune-desert and immune-excluded tumors were not characterized by a cordon of immunosuppressive macrophages or Tregs, as reported in cervical cancer and colorectal cancer.^35 36^ On the contrary, most HNSCCs were characterized by a remarkably high number of CD163+macrophages and Tregs throughout the tumor. CD163+macrophages as well as Tregs had the highest tendency to infiltrate the tumor center where they likely suppress antitumor immune functions or promote tumor progression. We noticed a relatively short distance from CD8+ and CD4+ T helper cells to Tregs and CD163+macrophages, indicating cellular interaction. In line with this, Feng *et al* showed in 119 oral cancers that FoxP3+cells located closely by CD8+T cells correlated with worse overall survival,^8^ suggesting that Tregs in close proximity exert greater suppression on CD8+T cell antitumor immune responses. The immunotypes were based on CD8+T cell infiltration; however, infiltration of other immune cells was observed in some of the immune-desert and immune-excluded tumors. This raises the question of whether the field needs to change this nomenclature to CD8+T cell-desert and T cell-excluded.

CD163 was used in the current study to identify macrophages. While it is a widely recognized marker for the identification of protumorigenic M2-like macrophages, CD163 may also be present in other myeloid cells, such as suppressive dendritic cells.^37^ In concordance with this, we demonstrated, using publicly available single-cell RNA-sequencing data from Cillo *et al*^38^ that also in HNSCC, CD163 expression is not limited by M2-like macrophages (online supplemental figure 17, online supplemental table 4). While most CD163 expression was observed in macrophage clusters with described M2-like features, recognized by among others *MRC1*/CD206, *APOE*, *TREM2* and *C1QA-C*,^38 39^ some minor CD163 expression was noticed in other myeloid subclusters. While this emphasizes the complexity of phenotyping M2-like macrophages, it also suggests that the vast majority of CD163+quantified cells exhibit M2-like features and therefore are most likely protumorigenic macrophages.

We were able to link the immune cell topography to the secretome of matched tumors for a subset of the cohort (11 resection specimens). Cytokine levels were higher in fully infiltrated tumors (as indicated by CD8+T cells in the tumor center and within tumor fields) compared with tumors with primary infiltrates in the tumor stroma, at the tumor border, or those with no CD8+T cell infiltration at all. Furthermore, as Tregs express CC chemokine receptor 4 (CCR4), the ligand for CCL17,^26 40^ it was not unexpected to find a positive correlation between Treg densities and CCL17 levels in the secretome of matched tumors. Still, this had not been described yet in the context of head and neck cancers. On the other hand, Treg densities did not correlate with CCL20 levels, while they are described to be associated with increased in vitro Treg migration towards head and neck cancer cell lines.^41^ Also, what should be noted is that 92 proteins were included in the assay. CCL22 for instance, which can also bind to CCR4 on Tregs,^26 40^ and has been described to positively correlate with Treg migration,^42^ was not included in the assay. Since this is an exploratory study with a small patient group for which we had spatial data as well as secretome data, these secretome results should be interpreted with caution.

As previously reported,^12 21 29 43^ HPV-positive OPSCC were found to have higher B-lymphocyte and T-lymphocyte levels compared with HPV-negative OPSCC. For other HNSCC anatomical sites, HPV is not routinely being tested. Therefore, we assumed them to be HPV-negative. Within OCSCC, Nauta *et al* demonstrated that only 21 out of 1,069 (2.2%) appeared HPV-positive without clinical significance.^44^ In the same line, for LSCC HPV seems not of clinical significance.^45^,^47^ The study of Patel *et al* suggests that HPV testing could be of relevance in HSCC.^48^ Additional studies are warranted to clarify whether HPV testing is relevant in other anatomical sites next to OPSCC.

Approximately 80% of the recurrent/metastatic HNSCCs are resistant to ICI targeting the anti-PD-1/programmed death-ligand 1 (PD-L1) axis.^4 5^ There are many ways for the tumor to escape from antitumor immunity, such as directly suppressing antitumor immunity, hindering immune cell trafficking, hampering immune recognition by downregulating major histocompatibility complex class I molecules, or orchestrating a suppressive TIME.^49 50^ Protumorigenic macrophages play a central role in the latter. By way of illustration, Zhang *et al* showed that messenger RNA PD-L1 expression correlated with high CD206 expression in a cohort of 112 patients with LSCC.^51^ Specifically, they showed in vitro that tumor cells promote differentiation of macrophages into IL-10-producing macrophages, which in turn induced immune suppression by PD-L1 expression in tumor cells.^51^ In addition, protumorigenic macrophages themselves also express PD-L1, and by binding PD-1, they are able to suppress among others CD8+T cells.^52^ Noticing the high densities of CD163+macrophages in the current study, it is plausible that CD163+macrophages play a central role in resistance to ICI in head and neck cancers. It is likely that their suppressive mechanisms go beyond the PD-L1/PD-1 axis; otherwise, one might expect a higher efficacy response rate to anti-PD-(L)1 ICIs. It must be noted that in the neoadjuvant setting, treating locally-advanced HNSCC prior to surgery, higher overall response rates to anti-PD-(L)1 ICI have been reported compared with the recurrent/metastatic HNSCC setting.^53^ For future direction, it will be relevant to perform comprehensive spatial analyses of the TIME in primary HNSCC treated with ICI in a neoadjuvant setting (in a clinical trial) as well as in recurrent/metastatic HNSCC specimens treated with ICI. However, obtaining resection specimens in that setting will be challenging, and different metastatic sites may encompass a different TIME and response to treatment.^54^

Altogether, the current study comprehensively characterized the spatial TIME of HNSCCs using a unique data set of primarily surgical resection specimens. A different immune cell topography was perceived when comparing surgical resections from distinct head and neck anatomical sites. OCSCC had the highest density of Tregs compared with hypopharynx and LSCC. In addition, we also noticed dissimilarities in the location of cells relative to each other.

## supplementary material

10.1136/jitc-2024-009550 online supplemental file 1

10.1136/jitc-2024-009550 online supplemental figure 1

10.1136/jitc-2024-009550 online supplemental table 1

10.1136/jitc-2024-009550 online supplemental table 2

10.1136/jitc-2024-009550 online supplemental table 3

10.1136/jitc-2024-009550 online supplemental table 4

## Data Availability

Data are available upon reasonable request.
