# Supplementary material for: Immune cell topography of head and neck cancer
Source: J Immunother Cancer. 2024 Jul 24;12(7):e009550. doi: 10.1136/jitc-2024-009550 (PMC11284952; doi:10.1136/jitc-2024-009550)
Supplement: online supplemental file 1 [file jitc-12-7-s001.docx]

## SUPPLEMENTAL METHODS

### **Deparaffinization and antigen retrieval**

FFPE sections of 4 µm mounted on Superfrost plus adhesion microscope slides (Epredia, J1800AMNZ) were used for manual multiplex IHC staining. First, sections were deparaffinized according to standard protocol. In brief, the slides underwent a 60 minutes incubation at 60°C, followed by sequential immersion in xylene (two times 7.5 minutes each), 100% ethanol (5 minutes), and 97% ethanol (5 minutes). To neutralize endogenous peroxidase, a mixture of methanol and 0.3% hydrogen peroxidase (H_2_O_2_) was applied to the slides for 20 minutes. They were rinsed in 70% ethanol (5 minutes), followed by a rinse in ultrapure water (MilliQ, Merck). Subsequently, the tissue sections were fixed by submerging the slides in 10% neutral buffered formalin for two hours. Following fixation, the sections were washed using agitation (90 rpm) in ultrapure water and in Tris-buffered saline with 0.05% Tween-20 (Sigma-Aldrich, P2287) (TBST).

Next, the sections underwent heat-mediated antigen retrieval. They were placed into 400 ml antigen retrieval buffer comprising Tris and ethylenediaminetetraacetic acid (EDTA) (10mM Tris, 1 mM EDTA, pH9) and ProClin300 (Sigma-Aldrich, 48912-U). The sections were heated in the microwave until boiling (900 watt, 4.2 minutes, followed by 300 watt, 15 minutes). The slides were placed in ice water to cool down (15 minutes) and rinsed in ultrapure water using agitation (90 rpm). Using a hydrophobic pen (Vector Laboratories, ImmEdge, H-4000), the tissue section was encircled, followed by immersion in TBST with agitation (90 rpm).

### **Manual seven-color multiplex immunohistochemistry Opal staining**

Antigen-antibody binding was visualized with tyramide signal amplification (TSA)-Opal reagents using the Opal Polaris 7 Color Manual IHC Detection Kit (Akoya Biosciences, NEL861001KT). The protocol consisted of repetitive cycles, outlined in table 1. Incubation steps were performed in a dark, humidified chamber. Starting each cycle, the tissue sections were blocked using antibody diluent/block (PerkinElmer, ARD1001EA) for 10 minutes. Then, sections were incubated with a primary antibody and antibody diluent/block followed by rinsing in TBST (three times 2 minutes) using agitation (90 rpm). For primary antibody CD19 (cycle two), blocking consisted of a 30 minute incubation in 20% normal goat serum (Genetex, GTX3206) and antibody diluent/block. Next, the slides were incubated with a horseradish peroxidase-conjugated secondary antibody (PerkinElmer, ARH1001EA). To augment the CD19 reaction, slides were incubated with a biotin-labelled secondary antibody (Invitrogen, 31820), followed by a horseradish peroxidase-conjugated Streptavidin (Dako, P0397). Following secondary antibody incubation, the slides were rinsed in TBST (three times 2 minutes) using agitation (90 rpm). Next, a fluorophore-labelled tyramide, diluted in amplification diluent (PerkinElmer, FP1498) was added to generate the Opal signal amplification. After 10 minutes of incubation, the slides were washed in TBST (three times 2 minutes) using agitation.

Antibody stripping was conducted to eliminate the primary and secondary antibodies along with any nonspecific staining following each cycle. The sections were heated in TRIS-EDTA antigen retrieval buffer, as detailed above for the antigen retrieval. An exception was made after the fifth cycle, when a denaturation solution kit (Biocare Medical, DNS001L) was utilized for antibody stripping (supplemental table 1). Subsequently, staining of the cell nucleus was achieved by incubation with TBST containing 4 drops DAPI, according to manufacturer’s recommendations (PerkinElmer, FP1490) per ml (5 minutes), followed by washing in TBST and then ultrapure water. Finally, the sections were mounted using ProLong Diamond Antifade Mountant (Invitrogen, P36970) and covered with microscope cover glasses (VWR, ECN631-1575).

**Table 1. Seven-color multiplex immunohistochemistry steps and antibodies**

|  | Cycle 1 | Cycle 2 | Cycle 3 | Cycle 4 | Cycle 5 | Cycle 6 |
| --- | --- | --- | --- | --- | --- | --- |
| Primary Ab | CD44v6 | CD19 | CD3 | CD8 | FoxP3 | CD163 |
| Supplier | VUmc | Abcam | Dako | Dako | Abcam | Novusbio |
| Clone | U36 | EPR5906 | PAb | C8/144B | 236A/E7 | 10D6 |
| Product# | Produced in house | Ab134114 | A0452 | M7103 | Ab20034 | NB110-59935 |
| Dilution | 1/200 | 1/100 | 1/250 | 1/250 | 1/100 | 1/100 |
| Host+isotype | Mouse | Rabbit | Rabbit | Mouse IgG1 | Mouse IgG1 | Mouse IgG1 |
| Reaction | RT, 60 min | 4˚C, O/N | RT, 60 min | RT, 60 min | RT, 60 min | 4˚C, O/N |
| Secondary Ab | Goat-a-rabbit/mouse-HRP | Goat-a-mouse-biotin | Goat-a-rabbit/mouse-HRP | Goat-a-rabbit/mouse-HRP | Goat-a-rabbit/mouse-HRP | Goat-a-rabbit/mouse-HRP |
| Reaction | RT, 15 min | RT, 30 min | RT, 15 min | RT, 15 min | RT, 15 min | RT, 15 min |
| Substrate |  | Streptavidin-HRP |  |  |  | Opal TSA DIG, RT 10 min |
| Dilution |  | 1/300 |  |  |  |  |
| Reaction |  | RT, 10 min |  |  |  |  |
| Fluorophore | Opal690 | Opal570 | Opal620 | Opal Polaris 480 | Opal520 | Opal Polaris 780 |
| Dilution | 1/100 | 1/100 | 1/100 | 1/100 | 1/100 | 1/25 |
| Reaction | RT, 10 min | RT, 10 min | RT, 10 min | RT, 10 min | RT, 10 min | RT, 60 min |

Abbreviations: Ab antibody; PAb polyclonal antibody; IgG1 immunoglobulin G1; RT room temperature; O/N over night; HRP horseradish peroxidase.

### **Automatically five-color multiplex immunohistochemistry Opal staining**

Tissue was stained using a five-color panel as previously described (1). Antigen-antibody binding was visualized with TSA-Opal reagents (Akoya Biosciences). The protocol consisted of repetitive cycles, outlined in table 2.

**Table 2. Five-color multiplex immunohistochemistry steps and antibodies**

|  | Cycle 1 | Cycle 2 | Cycle 3 | Cycle 4 |
| --- | --- | --- | --- | --- |
| Primary Ab | CD103 | CD8 | Ki67 | Pan Cytokeratin |
| Supplier | Abcam | Abcam | Cell Signaling | Novus |
| Clone | EPR4166(2) | SP16 | D2H10 | AE1/AE3 |
| Product# | Ab129202 | Ab101500 | 9027S | NBP2-29429 |
| Dilution | 1/2000 | 1/50 | 1/50 | 1/100 |
| Fluorophore | Opal620 | Opal520 | Opal570 | Opal690 |
| Dilution | 1/150 | 1/150 | 1/150 | 1/150 |

### **Single cell RNA-sequencing (scRNA-seq) analysis external dataset**

Public available scRNA-seq data of 18 HPV-negative head and neck squamous cell carcinoma (HNSCC) specimen were downloaded via Gene Expression Omnibus (GEO) (https://www.ncbi.nlm.nih.gov/geo/) from Cillo et al (2) (GSE139324), see Table 2 for details per specimen. R version 4.2.3 with Seurat package version 4.3.0 (3) and Harmony version 0.1.0 (4) were used for the analysis as previously described (5). Briefly, the count matrices were loaded for quality control and pre-processing followed by major cell annotation. Following, the myeloid cell cluster as isolated and DEG analysis was performed between clusters using FindMarkers function with the Wilcoxon rank-sum test. Markers with an average log-fold change of > .25, were considered differentially expressed. Features had to be present in at least 10% of the cells in the cluster and only features with a p-value of < .01 were selected (supplemental table 3). Derived p-values were corrected for multiple testing by the Benjamini-Hochberg method.

**Table 2. scRNA-seq external dataset of Cillo used for myeloid subclustering.**

| Dataset |  |  | Immune cells after filtering | Anatomical site |
| --- | --- | --- | --- | --- |
| Cillo et al. (2) | HNSCC_1_TIL | GSM4138111 | 1,298 | Oral Cavity |
| GSE139324 | HNSCC_2_TIL | GSM4138113 | 2,286 | Oral Cavity |
|  | HNSCC_3_TIL | GSM4138115 | 2,868 | Oral Cavity |
|  | HNSCC_4_TIL | GSM4138117 | 2,026 | Oral Cavity |
|  | HNSCC_5_TIL | GSM4138119 | 445 | Oral Cavity |
|  | HNSCC_6_TIL | GSM4138121 | 1,658 | Larynx |
|  | HNSCC_7_TIL | GSM4138123 | 2,333 | Oral Cavity |
|  | HNSCC_8_TIL | GSM4138125 | 917 | Oral Cavity |
|  | HNSCC_9_TIL | GSM4138127 | 5,097 | Oral Cavity |
|  | HNSCC_10_TIL | GSM4138129 | 1,383 | Oral Cavity |
|  | HNSCC_11_TIL | GSM4138131 | 2,687 | Oral Cavity |
|  | HNSCC_12_TIL | GSM4138133 | 1,905 | Oral Cavity |
|  | HNSCC_13_TIL | GSM4138135 | 1,806 | Larynx |
|  | HNSCC_14_TIL | GSM4138137 | 1,913 | Oral Cavity |
|  | HNSCC_15_TIL | GSM4138139 | 1,819 | Oral Cavity |
|  | HNSCC_16_TIL | GSM4138141 | 3,623 | Oral Cavity |
|  | HNSCC_17_TIL | GSM4138143 | 3,109 | Oral Cavity |
|  | HNSCC_18_TIL | GSM4138145 | 2,647 | Oral Cavity |
| Total |  |  | **39,820** |  |

## REFERENCES

1. Rajamanickam V, Ballesteros-Merino C, Samson K, Ross D, Bernard B, Fox BA, et al. Robust Antitumor Immunity in a Patient with Metastatic Colorectal Cancer Treated with Cytotoxic Regimens. Cancer Immunol Res. 2021;9(6):602-11.

2. Cillo AR, Kurten CHL, Tabib T, Qi Z, Onkar S, Wang T, et al. Immune Landscape of Viral- and Carcinogen-Driven Head and Neck Cancer. Immunity. 2020;52(1):183-99 e9.

3. Hao Y, Hao S, Andersen-Nissen E, Mauck WM, 3rd, Zheng S, Butler A, et al. Integrated analysis of multimodal single-cell data. Cell. 2021;184(13):3573-87 e29.

4. Korsunsky I, Millard N, Fan J, Slowikowski K, Zhang F, Wei K, et al. Fast, sensitive and accurate integration of single-cell data with Harmony. Nat Methods. 2019;16(12):1289-96.

5. Muijlwijk T, Nijenhuis D, Ganzevles SH, Brink A, Ke C, Fass JN, et al. Comparative analysis of immune infiltrates in head and neck cancers across anatomical sites. J Immunother Cancer. 2024;12(1).
