# Supplementary material for: Immune cell topography of head and neck cancer
Source: J Immunother Cancer. 2024 Jul 24;12(7):e009550. doi: 10.1136/jitc-2024-009550 (PMC11284952; doi:10.1136/jitc-2024-009550)
Supplement: online supplemental figure 1 [file jitc-12-7-s002.docx]

## SUPPLEMENTAL FIGURES


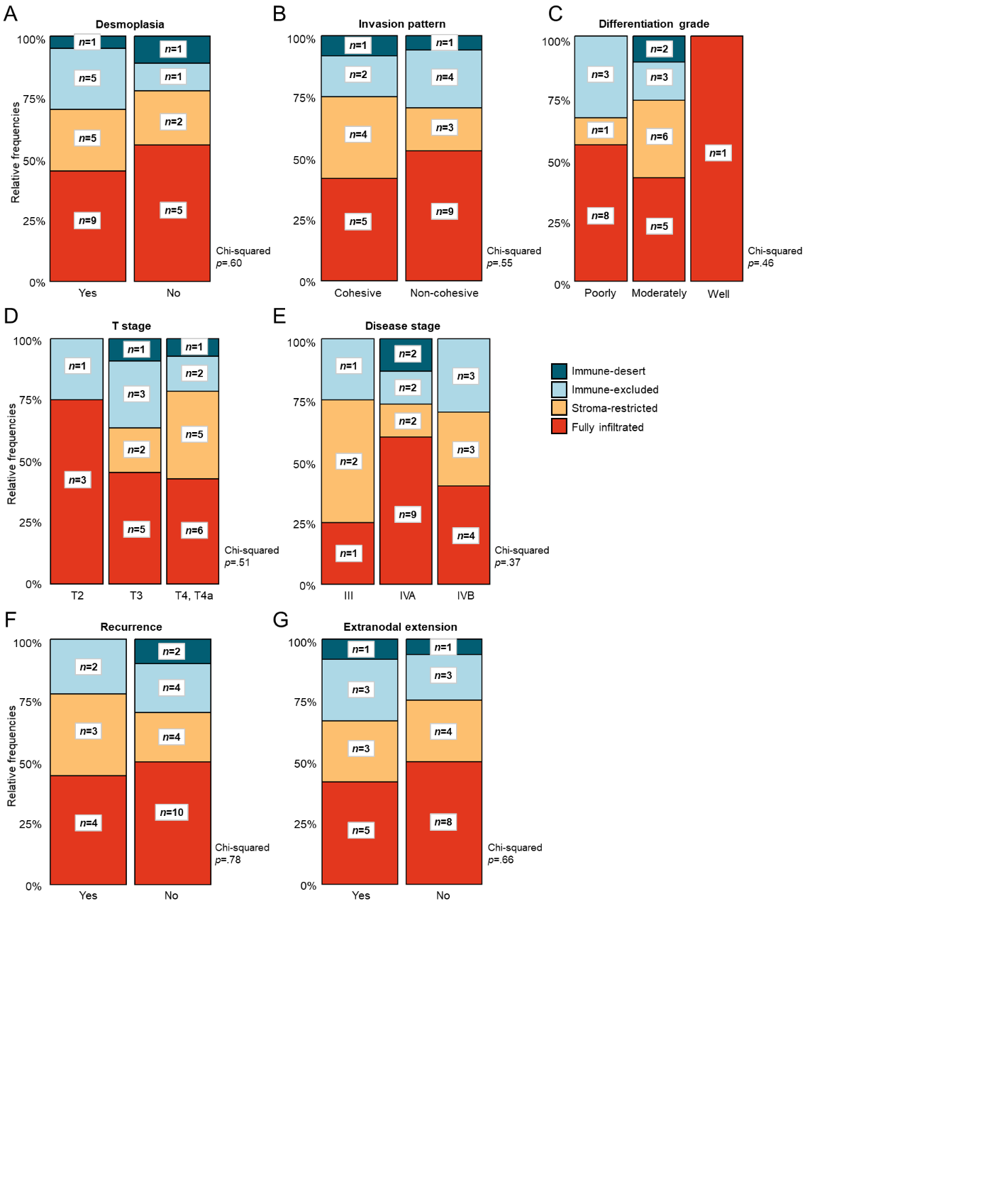


**Supplemental Figure 1. Histological and clinical characteristics across immunotypes of 29 HPV-negative head and neck squamous cell carcinoma (HNSCC) resection specimens. A-G)** Relative frequencies and number of specimens per category of **A)** presence of desmoplastic tissue, **B)** invasion pattern, **C)** differentiation grade, **D)** pathological T-stage and **E)** disease-stage according to TNM classification 8th edition, 2017, **F)** presence of recurrence and **G)** extranodal extension. Chi-squared tests were performed to obtain p-values. Fully infiltrated immunotype compared to other immunotypes since groups were too small.


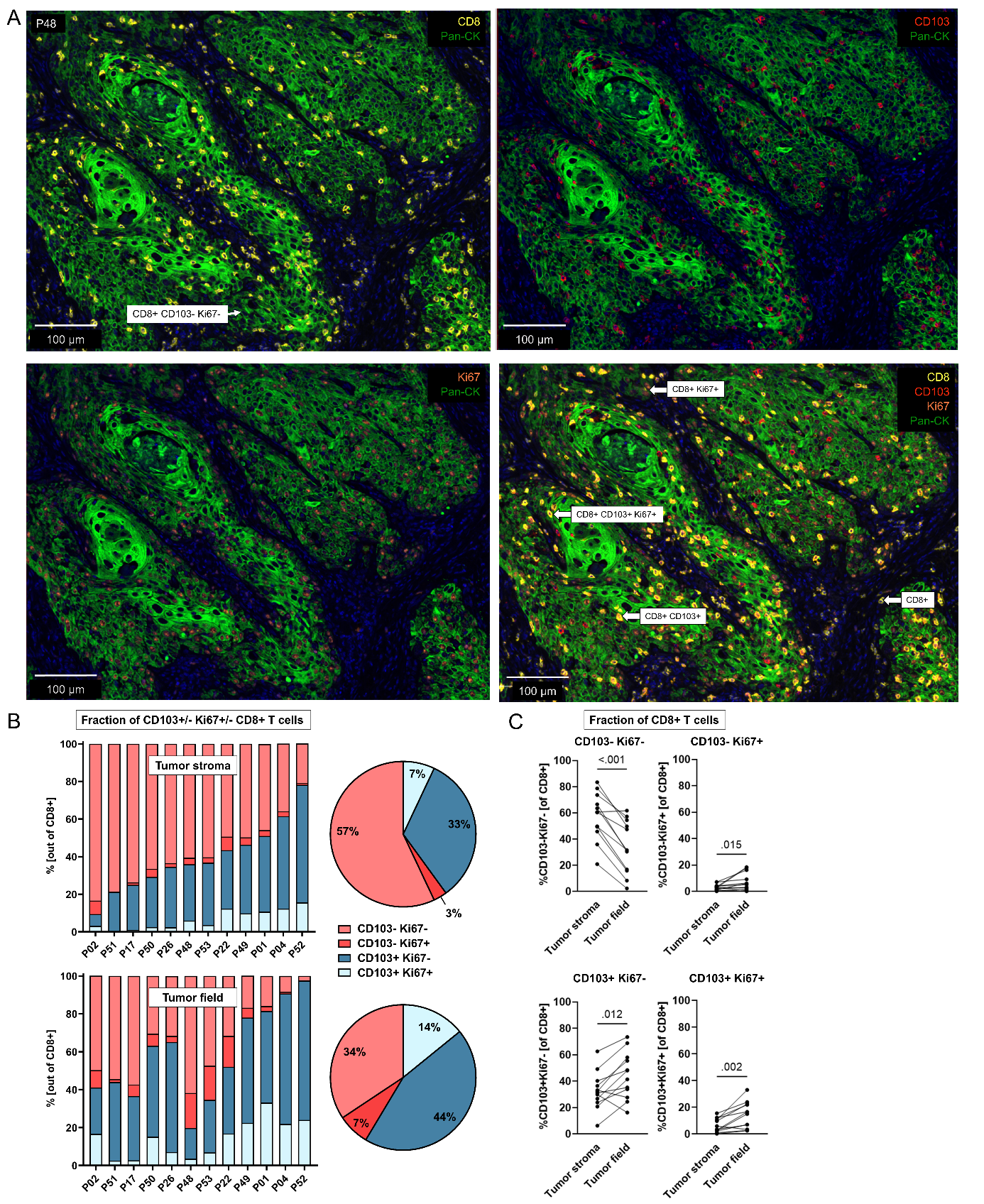


**Supplemental Figure 2. Characterization of cytotoxic CD8+ T cells within eleven HPV-negative head and neck squamous cell carcinoma (HNSCC) resection specimens.** **A)** Representative image of tumor (P48) stained with five-color multiplex IHC panel to distinguish CD103+ tumor-resident, CD103- infiltrating, Ki67+ proliferating and Ki67- non-proliferating CD8+ T cells. Tumor cells can be identified by their histomorphological appearance and as pan-cytokeratin (pan-CK)+. **B)** Fraction of CD103+/- Ki67+/- CD8+ T cells (y-axis) in twelve resection specimens (x-axis) in tumor stroma (upper panel) and tumor field (lower panel). Pie charts with average fraction in tumor stroma (upper pie chart) and tumor field (lower pie chart). **C)** Fraction of CD103+/- Ki67+/- CD8+ T cells (y-axis) in tumor stroma versus tumor field (x-axis), p-values obtained by paired non-parametric Wilcoxon tests.


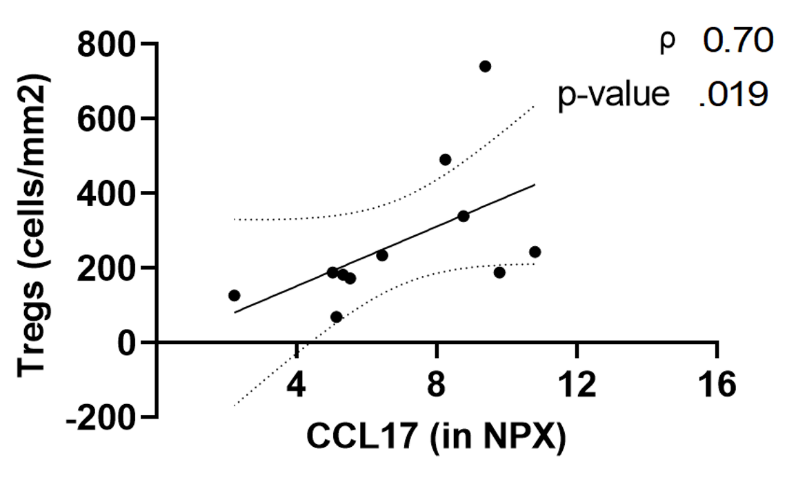


**Supplemental Figure 3. Correlation between immune cell densities in tumor area of eleven head and neck squamous cell carcinoma resection specimens and protein levels in overnight cultures of single cell suspensions from fresh matched tumor specimens.** Spearman’s correlation between Regulatory T cells (Tregs) in cells/mm^2^ (y-axis) and protein levels, in normalized protein expression (NPX) values (x-axis) of CCL17 measured in overnight cultures of 100,000 single cells from matched tumors.


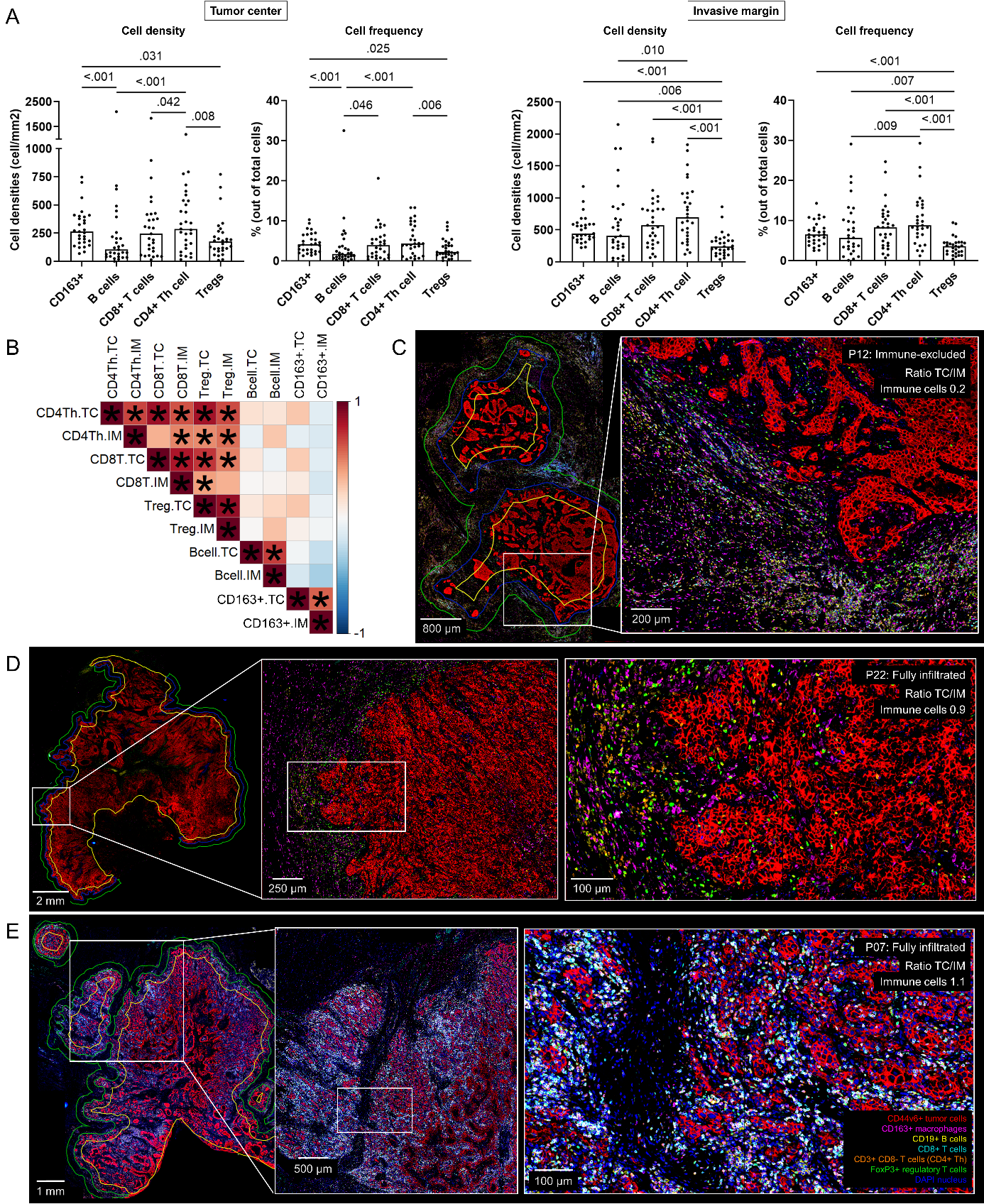


**Supplemental Figure 4. Immune cell densities and frequencies in 29 HPV-negative head and neck squamous cell carcinoma (HNSCC) resection specimens. A)** Densities (in cell/mm^2^) and frequency (out of total cells) in tumor center and invasive margin of CD163+ macrophages, B cells, CD8+ T cells, CD4+ T helper cells and regulatory T cells (Tregs). A paired non-parametric Friedman test was performed with uncorrected Dunn’s test to obtain p-values. Bars represent median values. **B)** Pearson correlation matrix with correlation coefficient from -1 (blue) to 1 (red) of immune cell densities in tumor center (TC) and invasive margin (IM) of 29 HPV-negative HNSCC resection specimens. P-values < .05 indicated with *. **C-E)** Representative images of tumors with **C)** higher density immune cells at the invasive margin compared to tumor center with a ratio of total immune cells in tumor center and invasive margin of 0.2 (P12), **D)** comparable density in the tumor center and at the border of the tumor with a ratio of total immune cells in tumor center to invasive margin of 0.9 (P22), and **E)** higher density of immune cells in the tumor center compared to the invasive margin with a ratio of total immune cells in tumor center to invasive margin of 1.1 (P07).

**
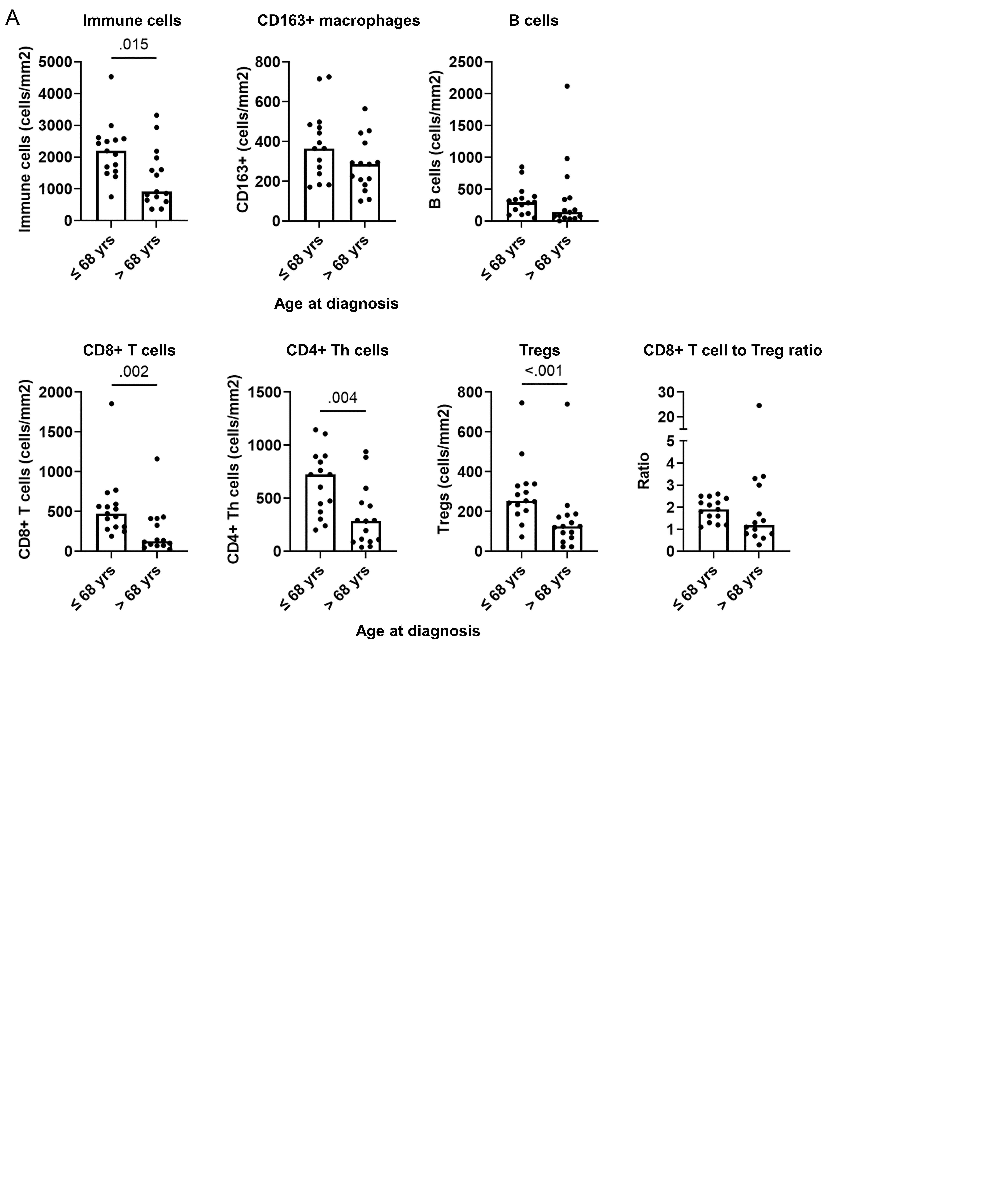
**

**
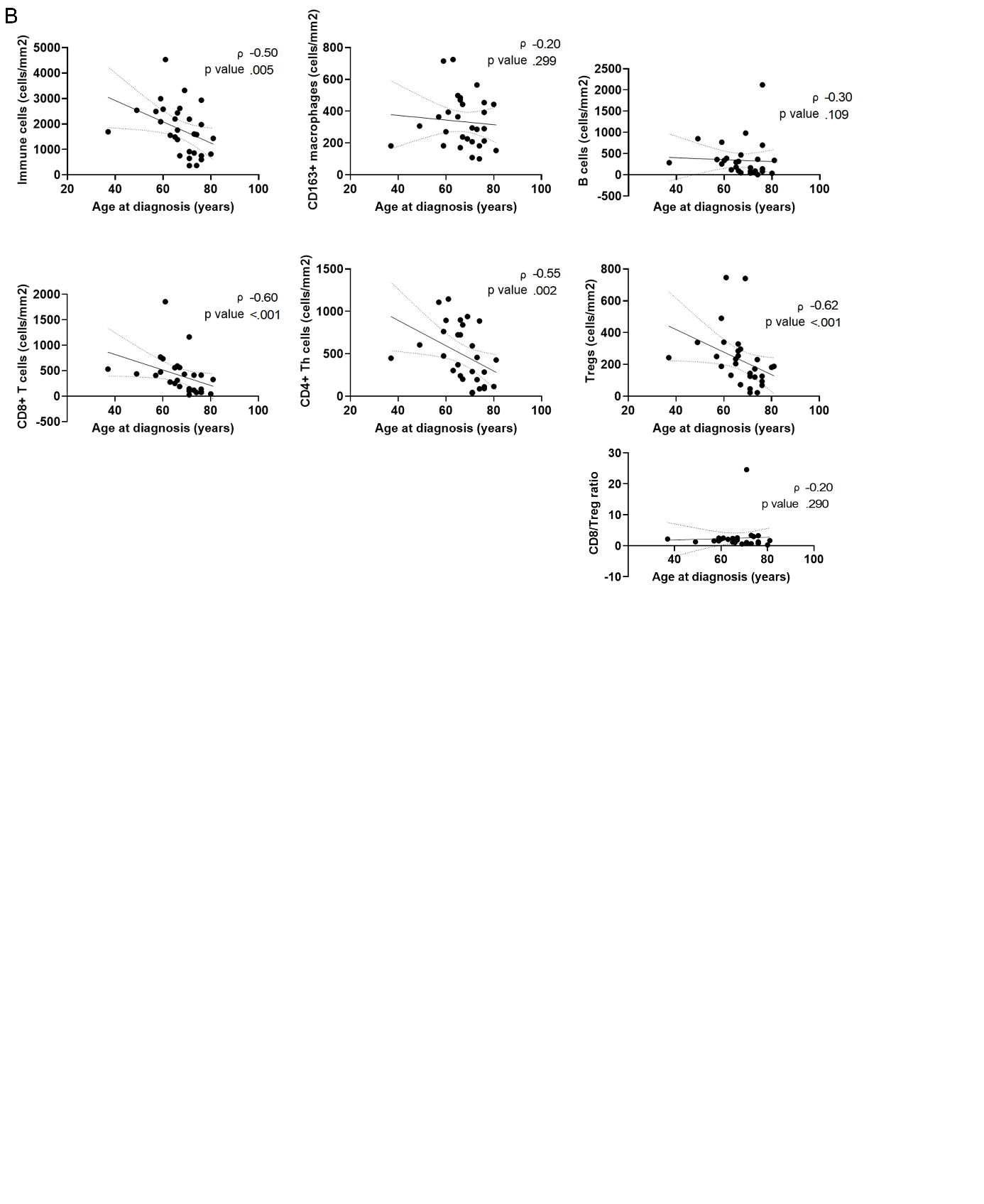
**

**Supplemental Figure 5. Immune cell densities in patients with HPV-negative head and neck squamous cells carcinoma (HNSCC) in regard to age at diagnosis. A)** Densities of immune cells, CD163+ macrophages, B cells, CD8+ T cells, CD4+ T helper (Th) cells, regulatory T cells (Tregs) in cells/mm^2^ and CD8+ T cell to Treg ratio (y-axis) between patients with the median age of 68 years or younger at diagnosis versus older patients (x-axis). Immune densities obtained from 30 resection specimens. P-values obtained by unpaired non-parametric Mann-Whitney tests, bars represent median values. **B)** Spearman correlation between immune cell densities as well as CD8+ T cell to Treg ratio (y-axis) and age at diagnosis in years (x-axis).


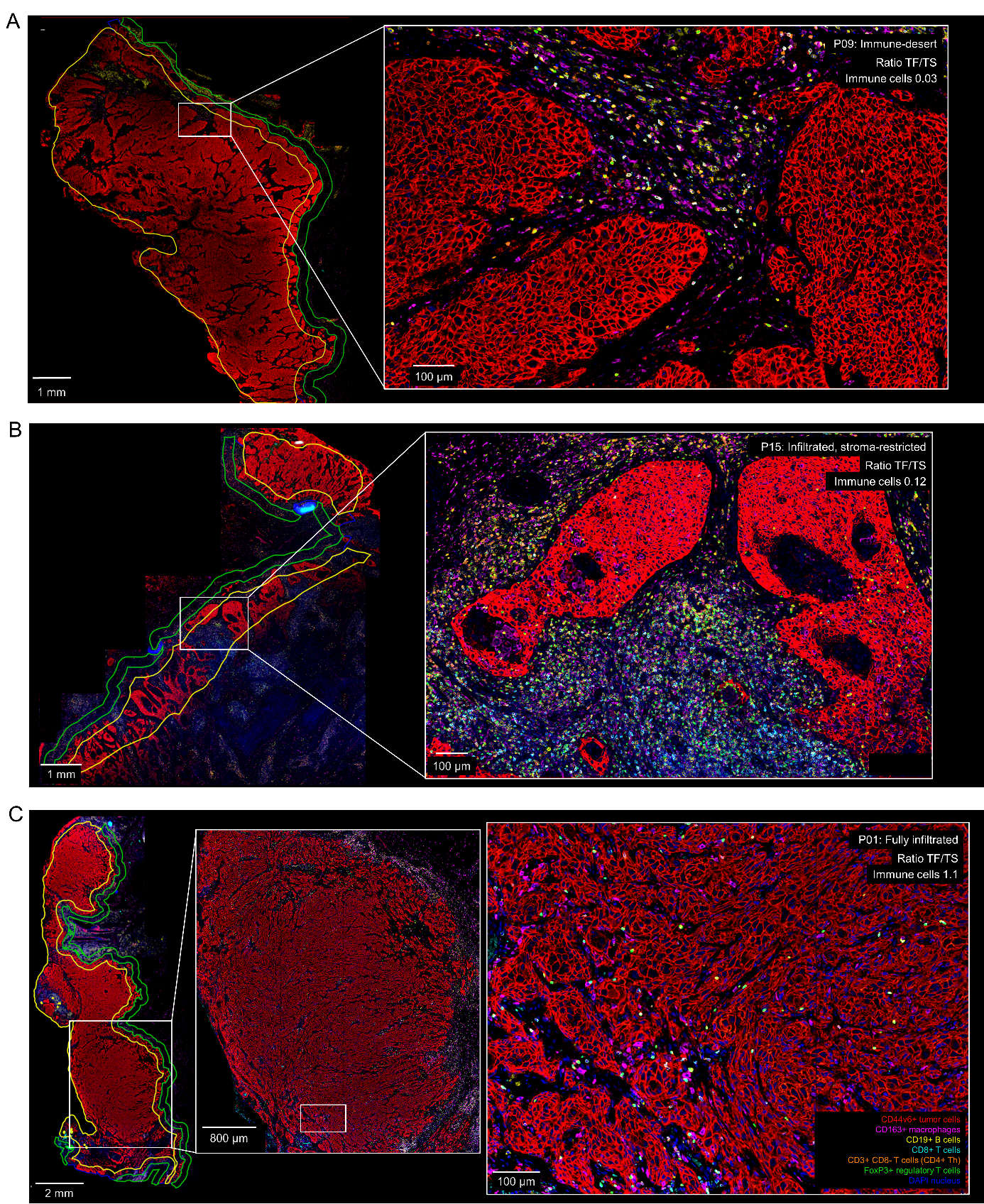


**Supplemental Figure 6. Representative images of HPV-negative head and neck squamous cell carcinoma (HNSCC) resection specimens. A-C)** Representative images of tumors with **A-B)** higher immune cell density in tumor stroma (TS) compared to tumor field (TF) with a ratio of total immune cells in tumor field to tumor stroma of 0.03 (P09) and 0.12 (P15) and **C)** higher density in tumor fields compared to tumor stroma with a ratio of total immune cells in tumor field to tumor stroma of 1.1 (P01).


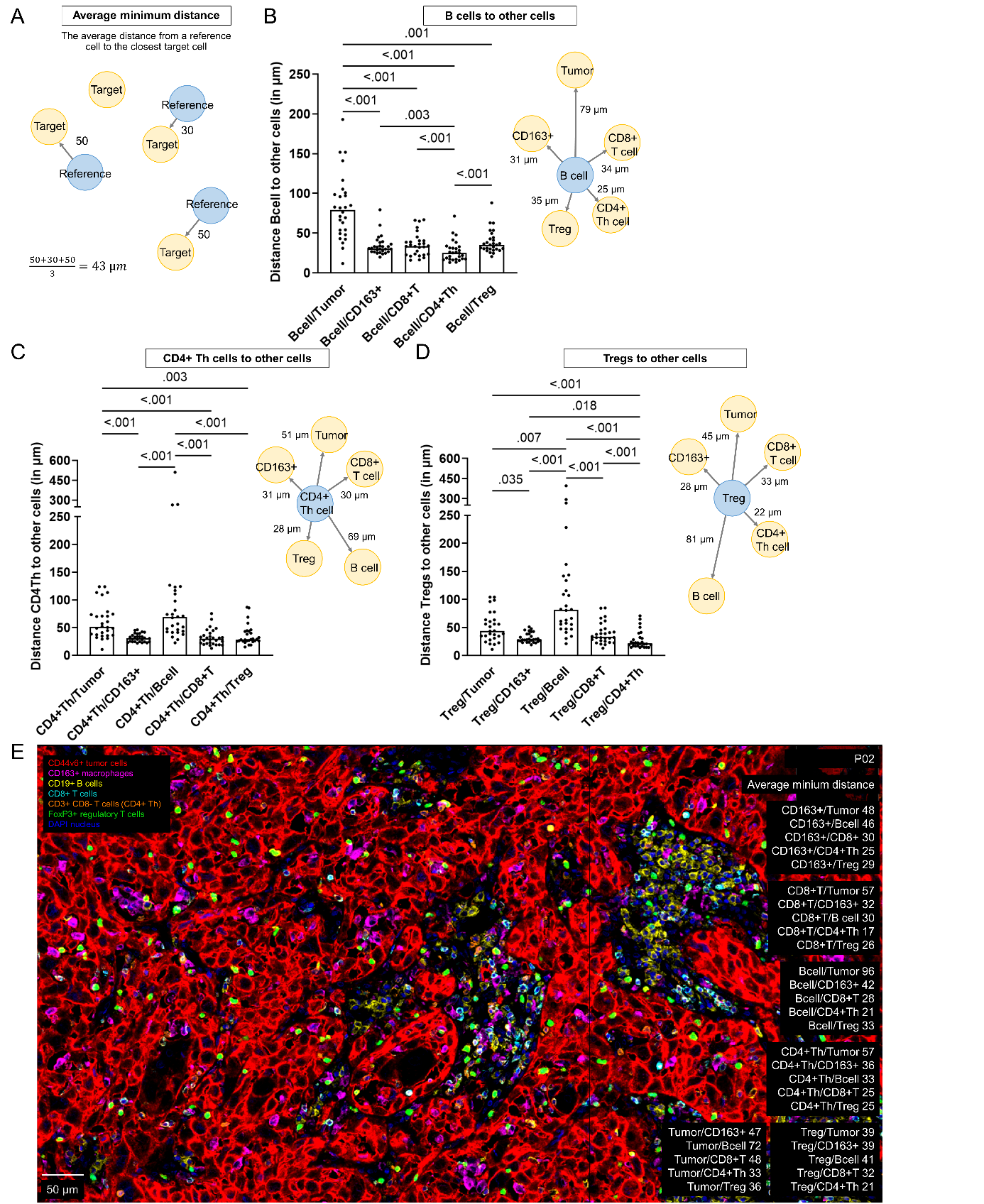


**Supplemental Figure 7. Average minimum distance (AMD) between cells in 29 HPV-negative head and neck squamous cell carcinoma (HNSCC) resection specimens. A)** Schematic overview of how the AMD from a reference cell (in blue) to the nearest target cells (in yellow) is being calculated. **B-D)** Distance (in µm, y-axis) of **B)** B cells, **C)** CD4+ T helper cells and **D)** Regulatory T cells (Tregs) to other cells in the tumor area (x-axis) in left panels. With a paired non-parametric Friedman test was performed with uncorrected Dunn’s test to obtain p-values and bars represent median values. Schematic overview of reference and target cells in right panel. **E)** Representative image of tumor with clustered B cells, CD163+ macrophages closest to tumor cells and T cells often localized by other T cells and M2-like macrophages with all AMD specified (P02).


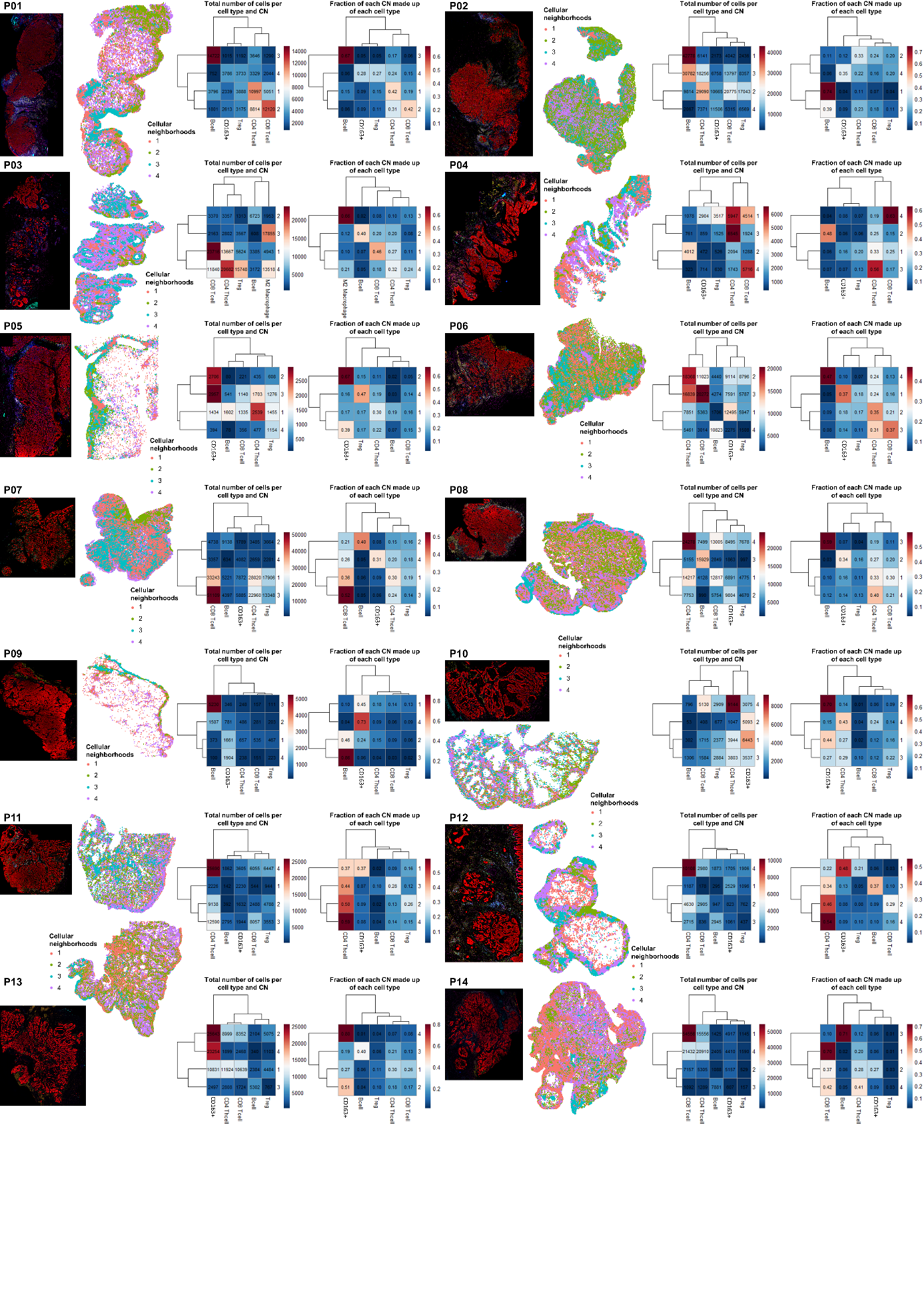


**Supplemental Figure 8. Immune cell neighborhood analysis for HPV-negative head and neck squamous cell carcinoma (HNSCC) resection specimens P01 to P14.** Neighboring cells in a radius of 50 µm were calculated using imcRtools (1). For each tumor, four neighborhoods were calculated. Representative images of tumors with animated map of immune cellular neighborhoods, a heatmap with total number of cells per neighborhood, and a heatmap with the fraction of each cell type per neighborhood (blue to red, y-axis) per cluster (x-axis).


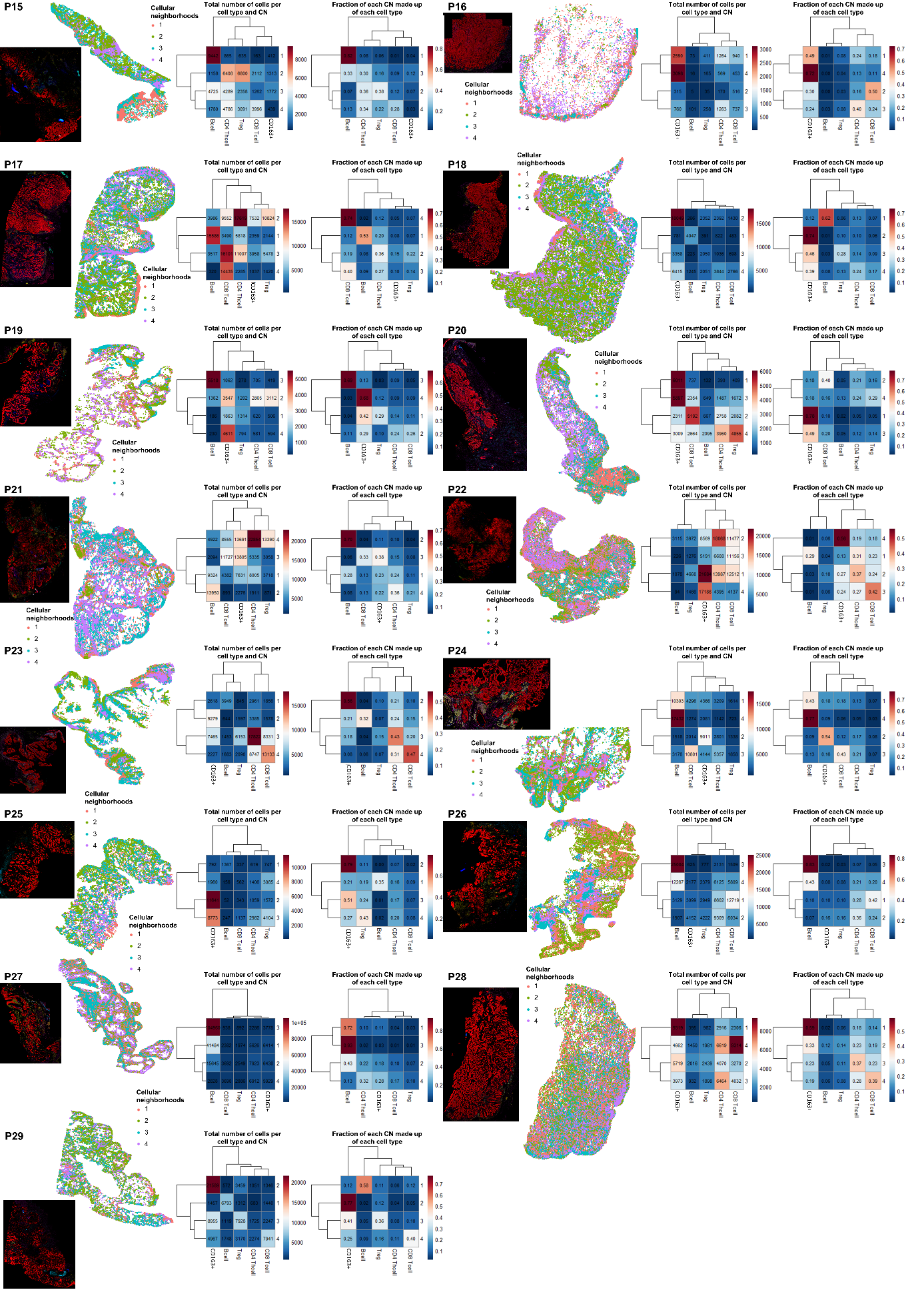


**Supplemental Figure 9. Immune cell neighborhood analysis for HPV-negative head and neck squamous cell carcinoma (HNSCC) resection specimens P15 to P29.** Neighboring cells in a radius of 50 µm were calculated using imcRtools (1). For each tumor, four neighborhoods were calculated. Representative images of tumors with animated map of immune cellular neighborhoods, a heatmap with total number of cells per neighborhood, and a heatmap with the fraction of each cell type per neighborhood (blue to red, y-axis) per cluster (x-axis).


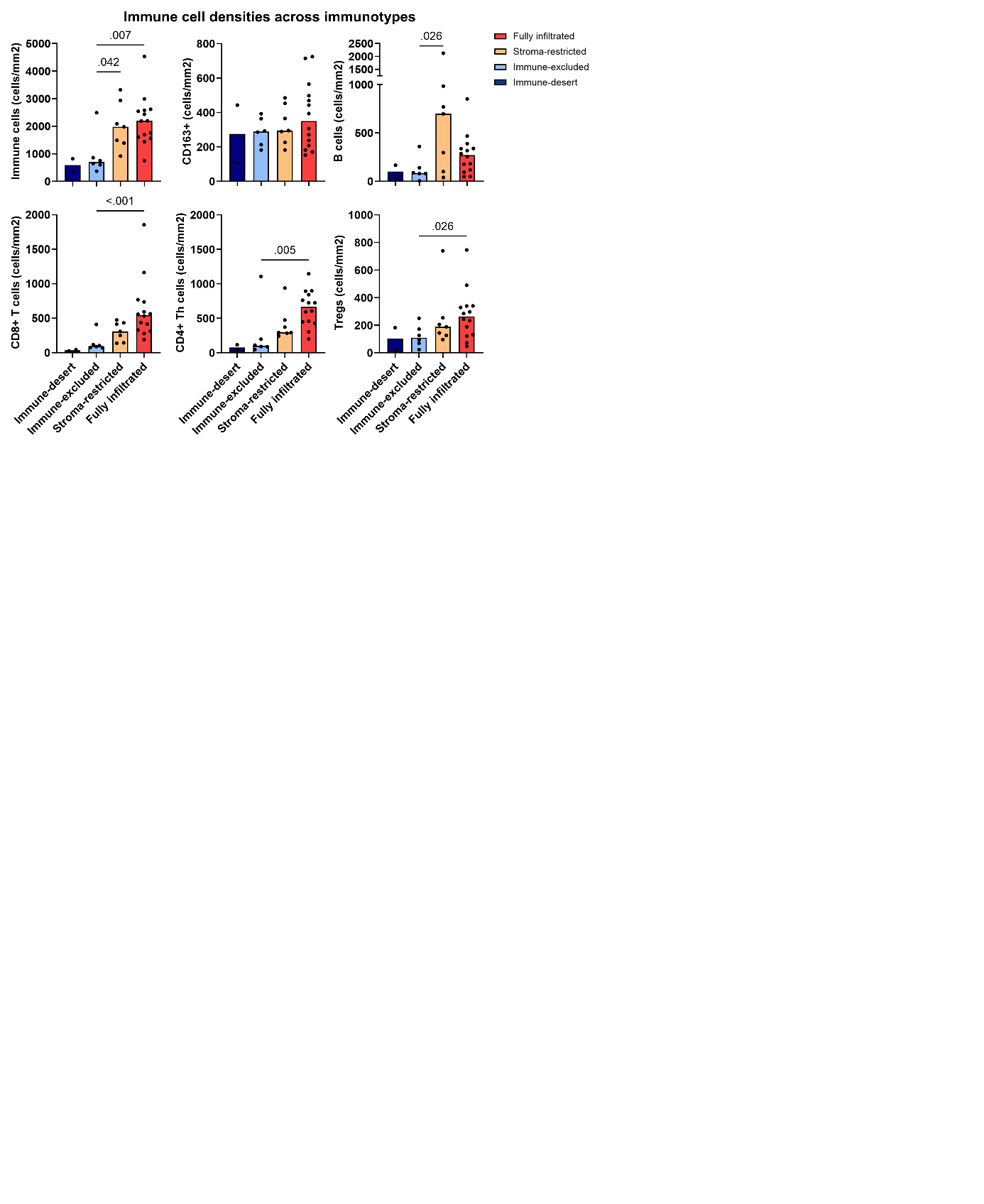


**Supplemental Figure 10. Immune cell densities across immunotypes of 29 HPV-negative head and neck squamous cell carcinoma (HNSCC) resection specimens.** Densities of immune cells, CD163+ macrophages, B cells, CD8+ T cells, CD4+ T helper (Th) cells and regulatory T cells (Tregs) in cells/mm^2^ (y-axis) in tumor area of immune-desert, immune-excluded, stroma-restricted and fully infiltrated tumors (x-axis). Bars represent median values, an unpaired non-parametric Kruskal-Wallis test with uncorrected Dunn’s test was performed to obtain p-values. Comparisons with immune-desert immunotypes were not feasible (*n*=2). Bars represent median values.


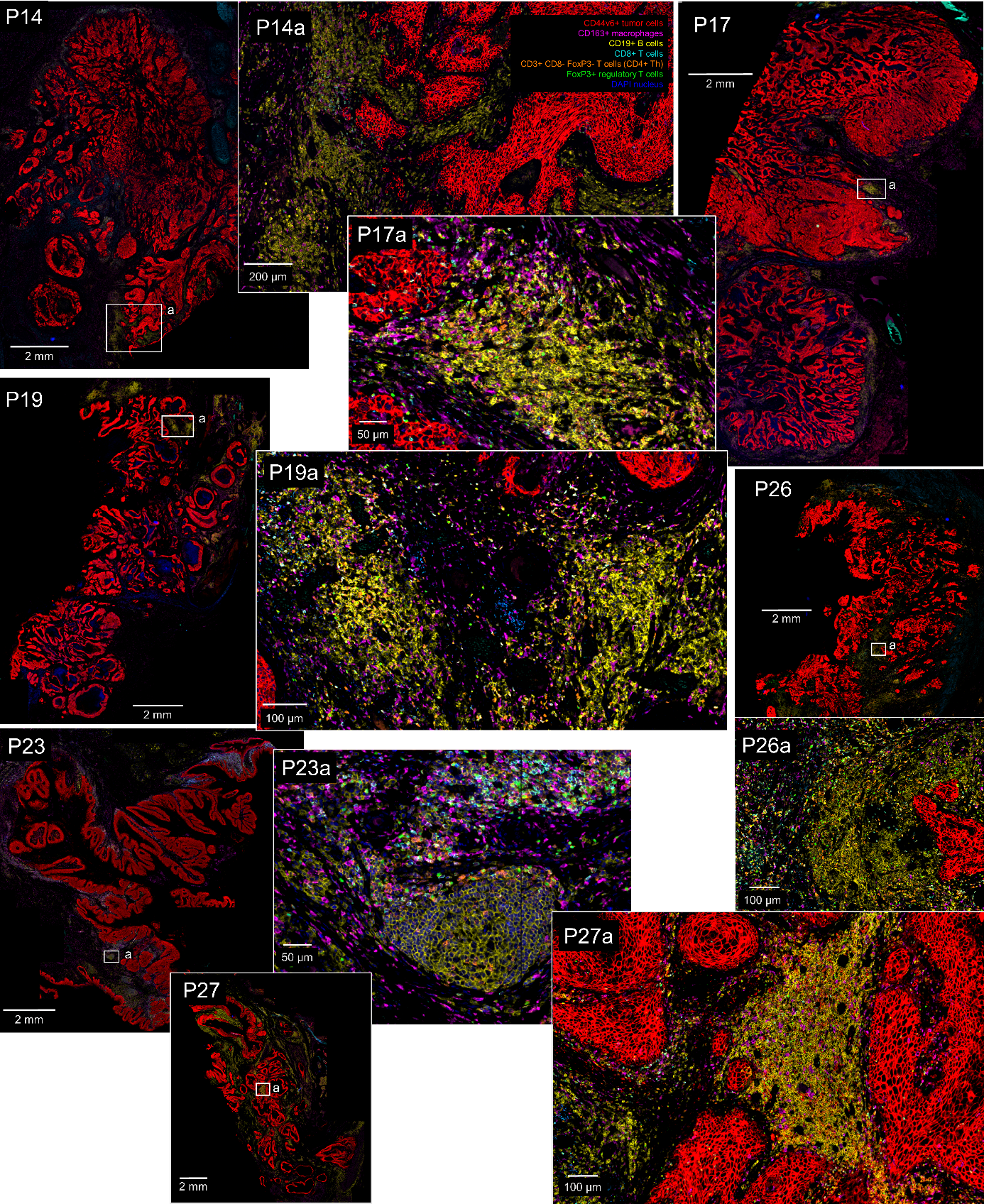


**Supplemental Figure 11. B cell immune cellular neighborhoods in HPV-negative head and neck squamous cell carcinoma (HNSCC) resection specimens.** Neighboring cells in a radius of 50 µm were calculated using imcRtools (1). For each tumor, four neighborhoods were calculated. Representative images of tumors P15, P18, P20, P24 and P28 with B cell aggregates zoomed in.


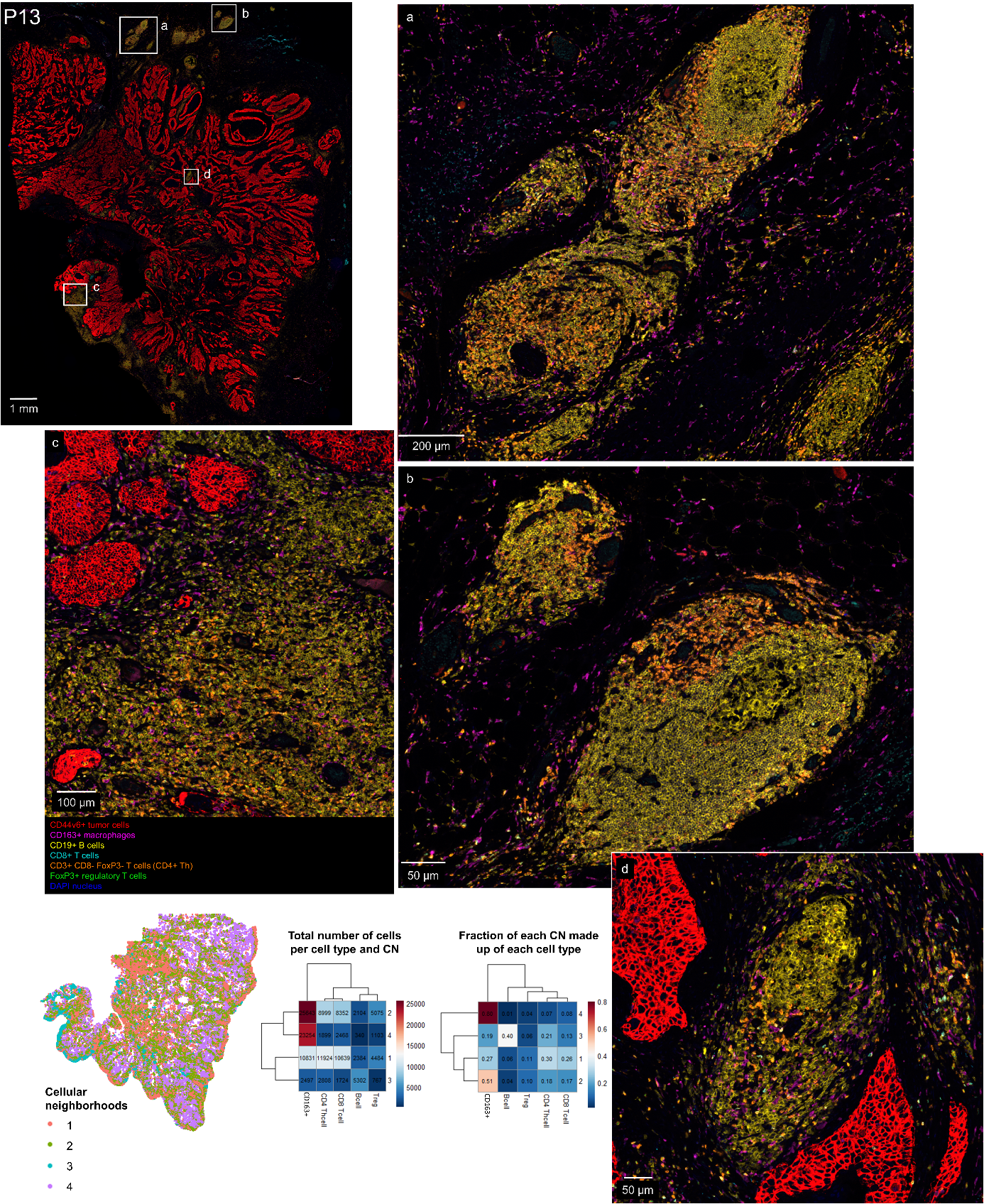


**Supplemental Figure 12. B cell immune cellular neighborhoods in HPV-negative head and neck squamous cell carcinoma (HNSCC) resection specimen P13.** Neighboring cells in a radius of 50 µm were calculated using imcRtools (1). For each tumor, four neighborhoods were calculated. Representative image of tumor P14 with B cell aggregates zoomed in, animated map of immune cellular neighborhoods, a heatmap with total number of cells per neighborhood and a heatmap with the fraction of each cell type per neighborhood (blue to red, y-axis) per cluster (x-axis).


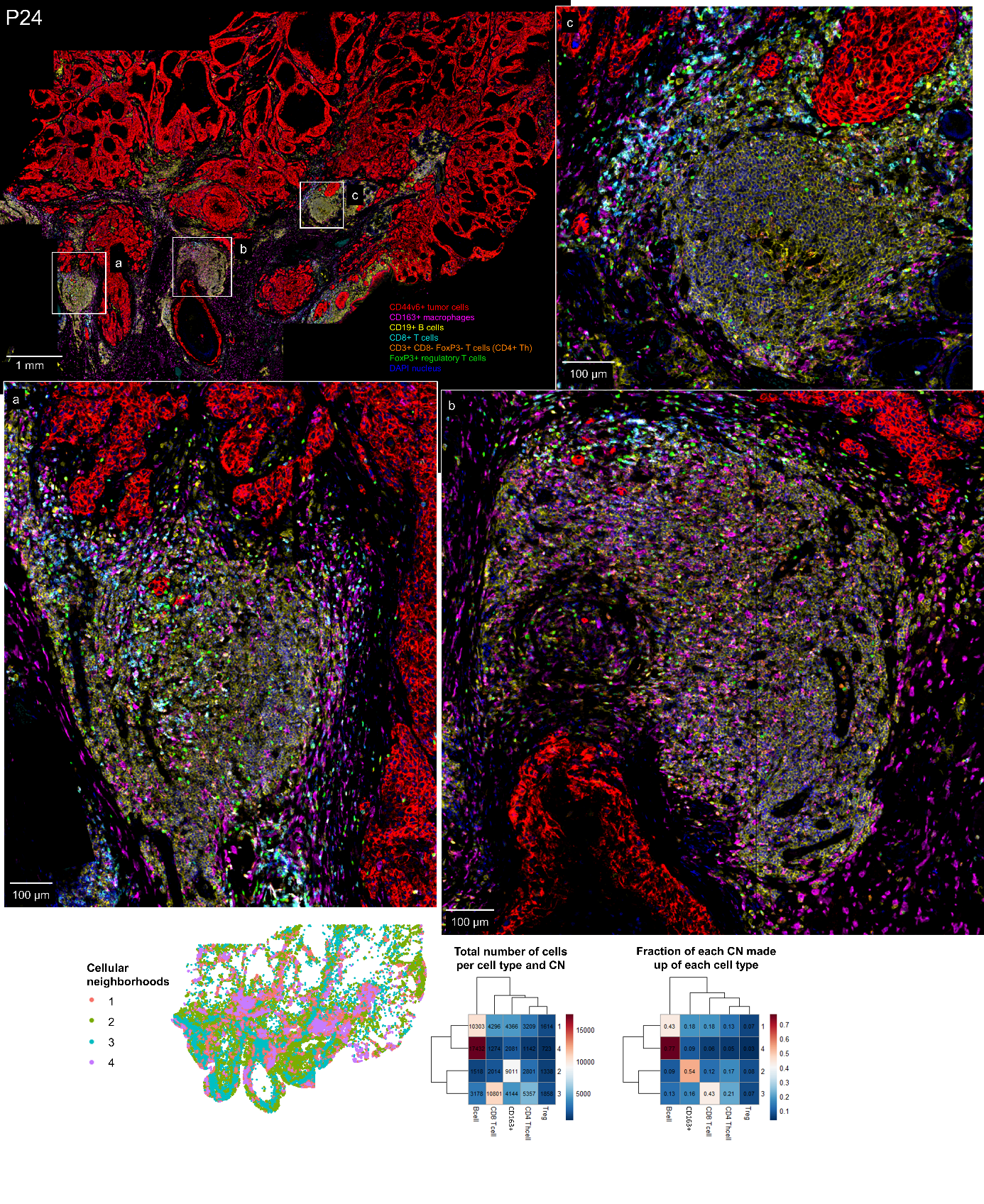


**Supplemental Figure 13. B cell immune cellular neighborhoods in HPV-negative head and neck squamous cell carcinoma (HNSCC) resection specimen P24.** Neighboring cells in a radius of 50 µm were calculated using imcRtools (1). For each tumor, four neighborhoods were calculated. Representative image of tumor P14 with B cell aggregates zoomed in, animated map of immune cellular neighborhoods, a heatmap with total number of cells per neighborhood and a heatmap with the fraction of each cell type per neighborhood (blue to red, y-axis) per cluster (x-axis).


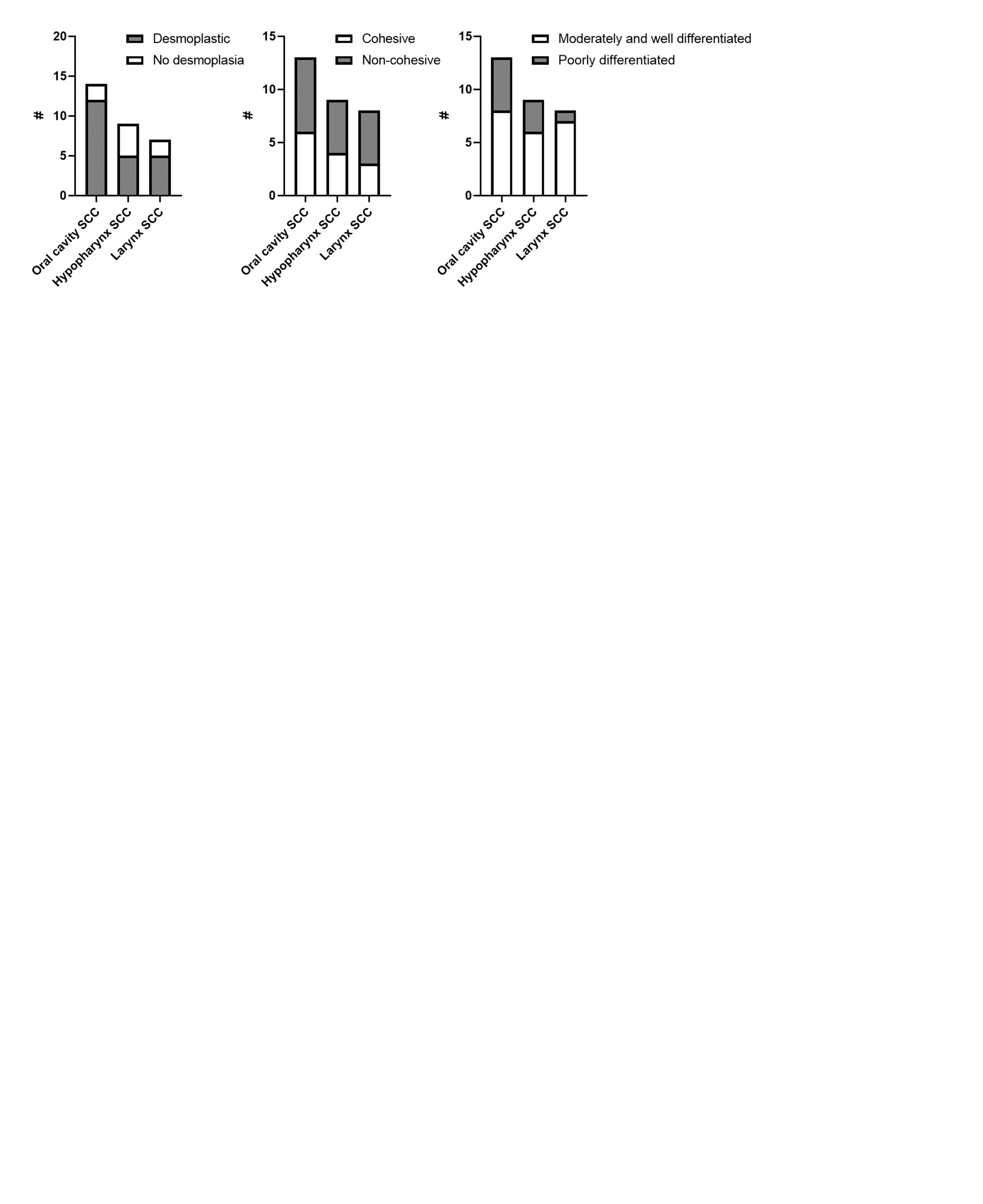


**Supplemental Figure 14. Histological parameters across HPV-negative head and neck squamous cell carcinomas (HNSCCs) from various anatomical sites.** Number of tumors (#, y-axis) with presence of desmoplastic tissue (dark grey) or not (white), non-cohesive (dark grey) or cohesive (white) invasion pattern, and poorly (dark grey) or moderately and well (white) differentiated tumors across anatomical sites (x-axis).


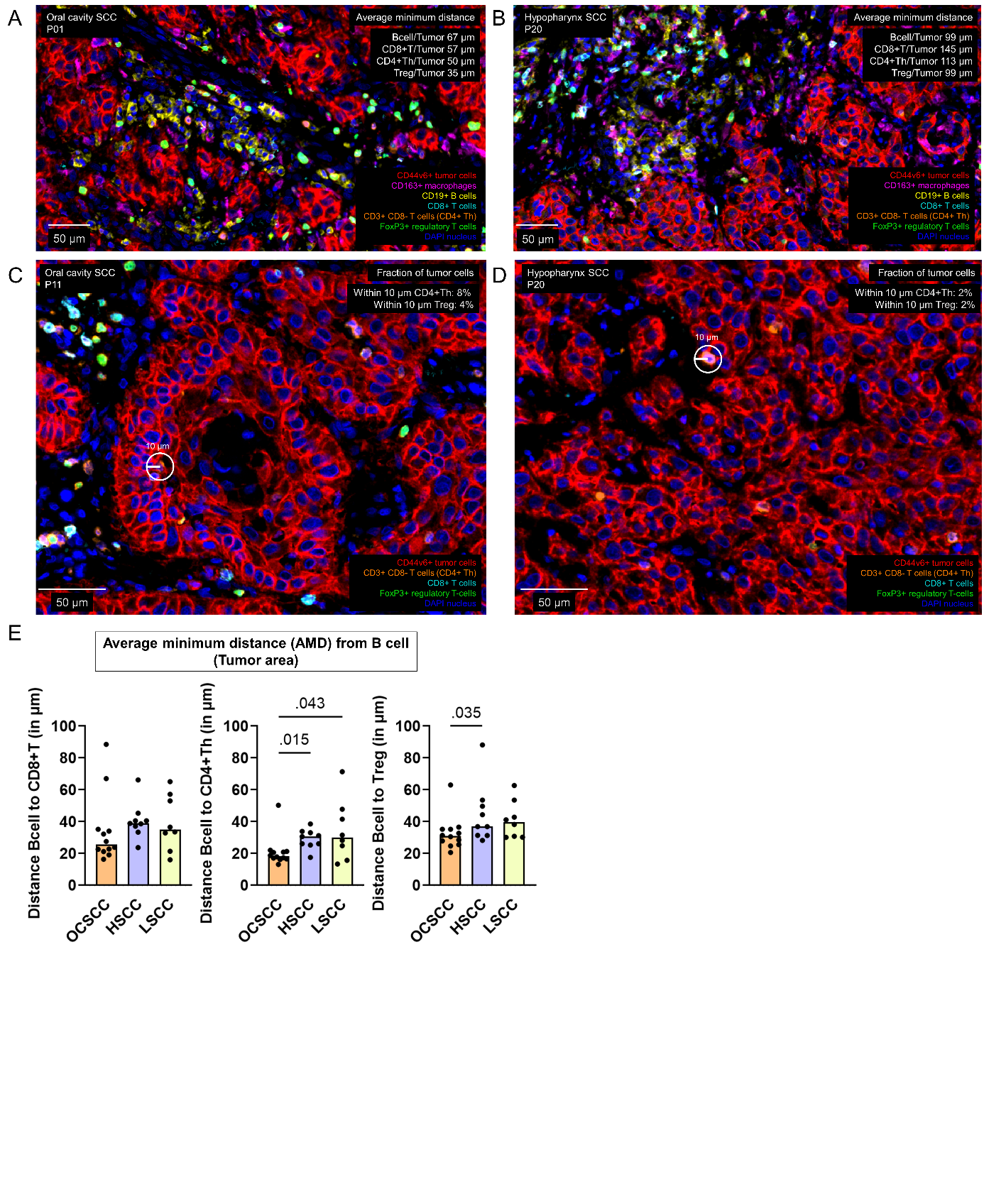


**Supplemental Figure 15. Distance and 10 µm radius analysis of 29 surgical resection specimens from oral cavity squamous cell carcinoma (OCSCC, *n*=12), hypopharynx SCC (HSCC, *n*=9), and larynx SCC (LSCC, *n*=8). A-B)** Representative images of **A)** OCSCC with longer average minimum distance (P01) and **B)** HSCC with closer average minimum distance (P20) of B and T cells to M2-like macrophages **C-D)** Representative images of **C)** OCSCC with higher percentage (P11) and **D)** HSCC with lower percentage (P20) of tumor cells within 10 µm radius of CD4+ T helper (Th) cells and regulatory T cells (Tregs). **E)** Average minimum distance from B cells to T cells (in µm, y-axis) across OCSCC, HSCC and, LSCC resection specimens (x-axis), p-values obtained by unpaired non-parametric Mann-Whitney tests, bars represent median values.


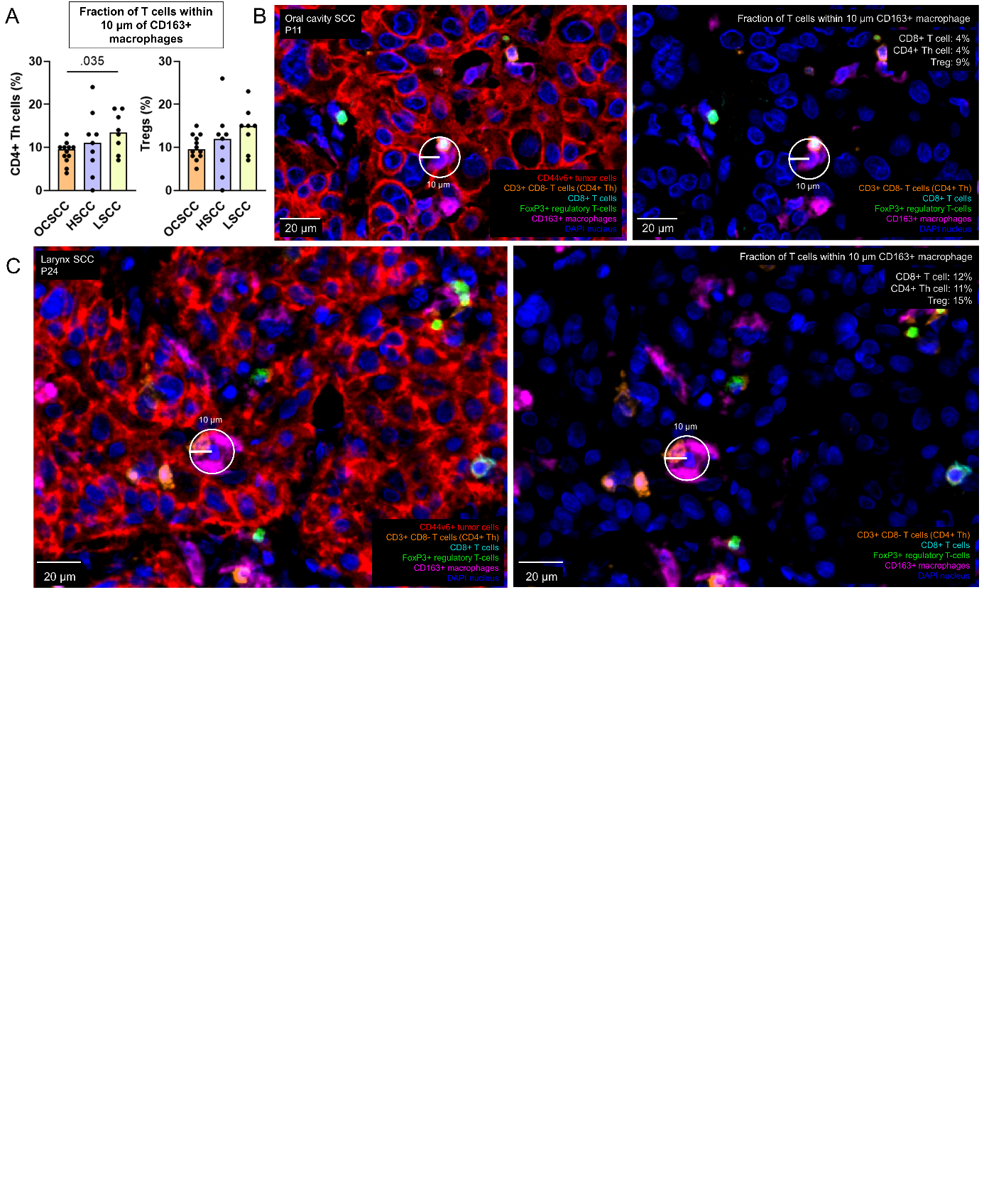


**Supplemental Figure 16. Radius analysis of 29 surgical resection specimens from oral cavity squamous cell carcinoma (OCSCC, *n*=12), hypopharynx SCC (HSCC, *n*=9), and larynx SCC (LSCC, *n*=8). A)** Fraction of T cells (y-axis) within 10 µm of M2-like macrophages in tumor center of OCSCC, HSCC, and LSCC resections. Unpaired non-parametric Kruskal-Wallis tests with uncorrected Dunn’s tests were performed to obtain p-values, bars represent median values. **B-C)** Representative images of **B)** OCSCC with lower percentage (P11) and **C)** LSCC with higher percentage (P24) of T cells within 10 µm radius of CD163+ macrophages.


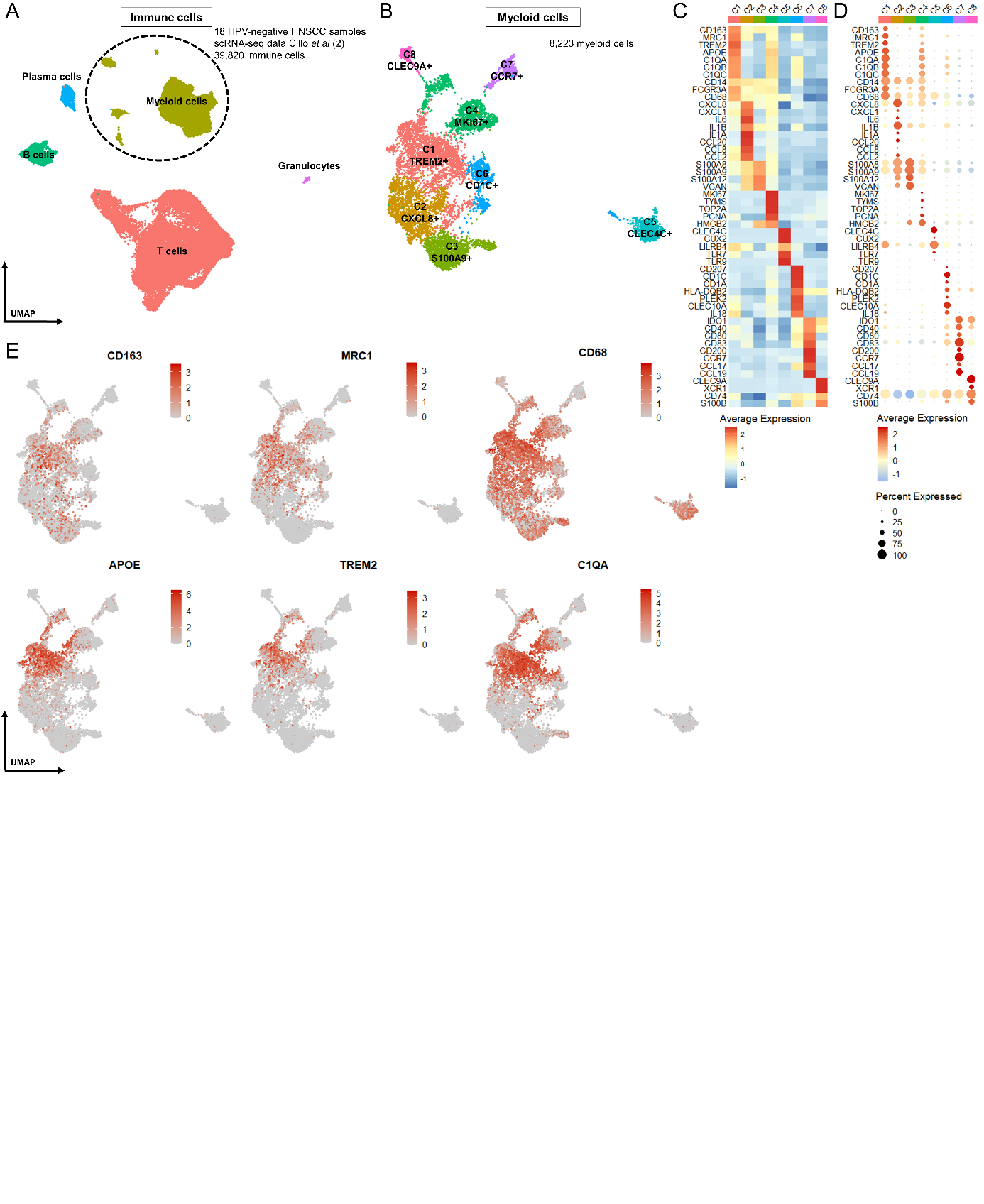


**Supplemental Figure 17. M2-like macrophage marker expression in myeloid cells from 18 HPV-negative head and neck squamous cell carcinomas using the external single cell RNA-sequencing (scRNA-seq) dataset of Cillo *et al* (2). A-B)** Uniform Manifold Approximation and Projection (UMAP) with **A)** Major cell type annotation of 39,820 immune cells. **B)** Subclustering of 8,223 myeloid cells. **C-D)** Average expression (from -1 in blue to 2 in red) subclusters C1 to C8 (x-axis) of various signature genes used for cell type annotation (y-axis) presented as **C)** a heatmap and **D)** a dotplot were the dots represent the percent expressed in that certain cluster. See supplementary table 4 for a list of the differentially expressed genes. **E)** mRNA expression of *CD163*, *MRC1*/CD206, *CD68*, *APOE*, *TREM2* and *C1QA* in myeloid cell subclusters visualized using a UMAP.

## REFERENCES

1. Windhager J, Zanotelli VRT, Schulz D, Meyer L, Daniel M, Bodenmiller B, Eling N. An end-to-end workflow for multiplexed image processing and analysis. Nat Protoc. 2023;18(11):3565-613.

2. Cillo AR, Kurten CHL, Tabib T, Qi Z, Onkar S, Wang T, et al. Immune Landscape of Viral- and Carcinogen-Driven Head and Neck Cancer. Immunity. 2020;52(1):183-99 e9.
